# Engineering Multi-field-coupled Synergistic Ion Transport System Based on the Heterogeneous Nanofluidic Membrane for High-Efficient Lithium Extraction

**DOI:** 10.1007/s40820-023-01106-5

**Published:** 2023-05-20

**Authors:** Lin Fu, Yuhao Hu, Xiangbin Lin, Qingchen Wang, Linsen Yang, Weiwen Xin, Shengyang Zhou, Yongchao Qian, Xiang-Yu Kong, Lei Jiang, Liping Wen

**Affiliations:** 1 CAS Key Laboratory of Bio-Inspired Materials and Interfacial Science, Technical Institute of Physics and Chemistry, Chinese Academy of Sciences, Beijing, 100190 People’s Republic of China; 2 School of Future Technology, University of Chinese Academy of Sciences, Beijing, 100049 People’s Republic of China; 3 Qingdao Institute of Bioenergy and Bioprocess Technology, Chinese Academy of Sciences, Qingdao, 266101 People’s Republic of China

**Keywords:** Nanofluids, Ion separation, Lithium extraction, Synergistic effect, Spent lithium-ion battery

## Abstract

The global carbon neutrality strategy brings a wave of rechargeable lithium‐ion batteries technique development and induces an ever-growing consumption and demand for lithium (Li). Among all the Li exploitation, extracting Li from spent LIBs would be a strategic and perspective approach, especially with the low energy consumption and eco-friendly membrane separation method. However, current membrane separation systems mainly focus on monotonous membrane design and structure optimization, and rarely further consider the coordination of inherent structure and applied external field, resulting in limited ion transport. Here, we propose a heterogeneous nanofluidic membrane as a platform for coupling multi-external fields (i.e., light-induced heat, electrical, and concentration gradient fields) to construct the multi-field-coupled synergistic ion transport system (MSITS) for Li-ion extraction from spent LIBs. The Li flux of the MSITS reaches 367.4 mmol m^−2^ h^−1^, even higher than the sum flux of those applied individual fields, reflecting synergistic enhancement for ion transport of the multi-field-coupled effect. Benefiting from the adaptation of membrane structure and multi-external fields, the proposed system exhibits ultrahigh selectivity with a Li^+^/Co^2+^ factor of 216,412, outperforming previous reports. MSITS based on nanofluidic membrane proves to be a promising ion transport strategy, as it could accelerate ion transmembrane transport and alleviate the ion concentration polarization effect. This work demonstrated a collaborative system equipped with an optimized membrane for high-efficient Li extraction, providing an expanded strategy to investigate the other membrane-based applications of their common similarities in core concepts.

**Supplementary Information:**

The online version contains supplementary material available at 10.1007/s40820-023-01106-5.

## Highlights

- The first construct of a multi-field-coupled synergistic ion transport system (MSITS) in Li^+^ extraction is proposed.
- Effectively suppress the ion concentration polarization effect of the ion-enrichment zone at the membrane interface.
- The MSITS equipped with heterogeneous membrane exhibited outstanding separation performance with Li^+^ flux of 367.4 mmol m^−2^ h^−1^ and Li^+^/Co^2+^ selectivity of 216,412, outperforming previous reports.

**Supplementary Information:**

The online version contains supplementary material available at 10.1007/s40820-023-01106-5.

## Graphical abstract

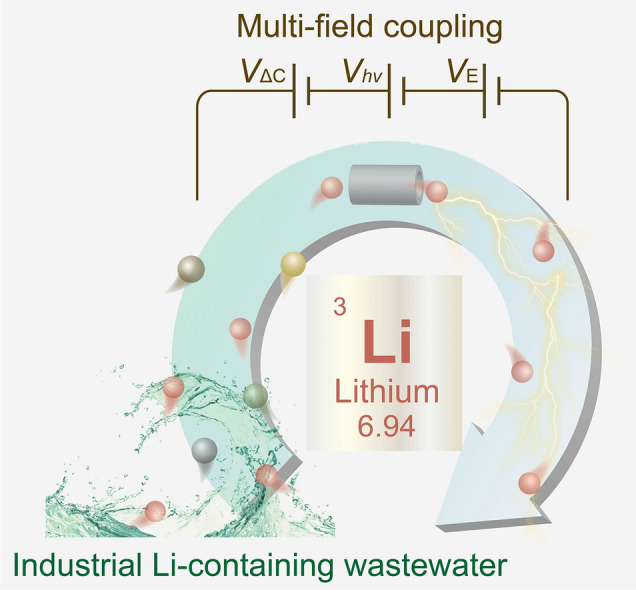

## Introduction

Lithium-ion batteries (LIBs) have become an indispensable energy storage system with wide applications, especially in portable electronic devices [1–4], electric vehicles, and power stations, which result in soaring demand for global lithium consumption as well as a surge of end-of-life LIBs [5–7]. Threatened by the uneven regional distribution of Li resources and the ever-growing environmental crisis, recycling spent LIBs is expected to reduce production costs and alleviate resource consumption, which remains an enormous challenge [8–10]. Li resource extraction and recovery, which calls for efficient and eco-friendly methods, is key to the development of sustainable energy and environmental protection [11, 12]. Over the past years, many approaches have been developed to extract Li including electrodialysis [13–15], solvent extraction [16], ion-sieve adsorption [17], solar evaporation precipitation [18], and electrochemical methods [19–21]. Separation technology based on the nanofluidic membrane is considered a promising and eco-friendly alternative for Li extraction, owing to the advantages of tunable ion transport and continuous operation [22–26]. Nanofluidic membranes, as advanced platforms integrating sufficient nanochannels and adjustable host–guest chemistries, exhibit a wide range of confined ion transport properties such as ion selective transport [27–29]. In particular, integrating Li^+^-selective nanofluidic membranes with well-designed transport systems can switch the trade-off between selectivity and flux positively, achieving improved Li^+^ extraction performance.

As known, the biological ion channel is not only capable of ultra-high ion selectivity and ion flux but also responds intelligently to environmental stimuli, allowing it the tunable ion transport properties under stimuli [30, 31]. Biomimetic artificial nanofluidic has been explored to regulate ion transport performance for wide applications such as energy conversion by applying external fields (e.g. light [32–34], heat [35], and force [36]). The external fields exerting at the systems may provide additional driven force for the ion transmembrane movement and hydrodynamic convection at the membrane interface, alleviating the degree of ICP in nanofluidic membranes during ion extraction, promising efficient recovery of resources [37]. Nevertheless, the current system emphasizes the effect of individual fields on confined ion transport, while ignoring the cooperative effect of multiple external fields. Transmembrane ionic transport promoted by physical fields has been found to promise efficient resource recovery. In addition to constructing nanofluidic membranes with controllable ion transport and stimuli responding, the collaboration of optimized membranes with multi-external fields needs to be considered to achieve enhanced ion transport.

**Fig. 1:**
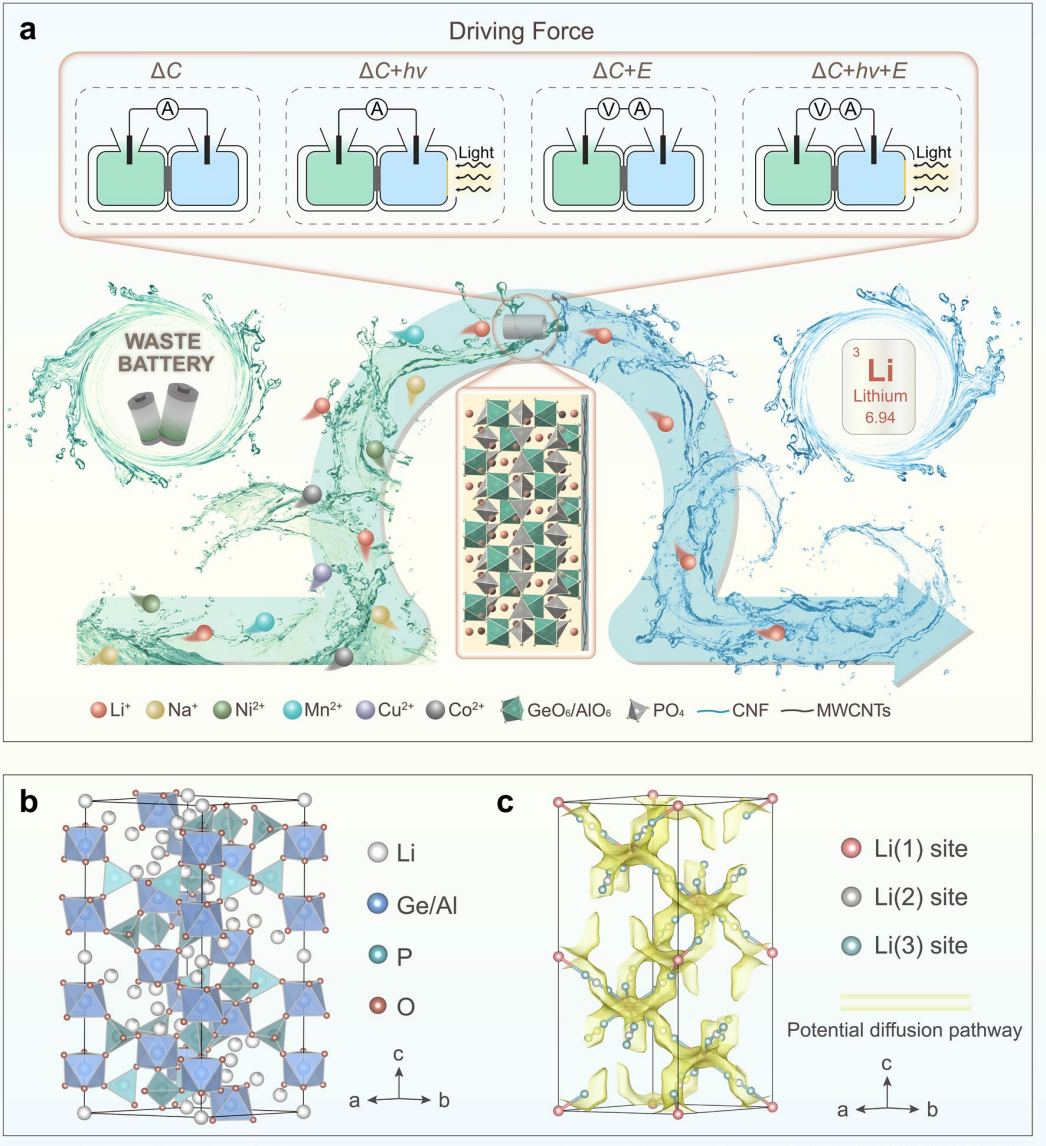
Setup of the MSITS. **a** Schematic representation of MSITS based on heterogeneous membrane (HM). The HM consists of LAGP and MWCNTs/CNF layers. Four external fields applied to the system include concentration gradient field (Δ*C*), concentration gradient field coupled with light-induced heat field (Δ*C* + *hv*), concentration gradient field coupled with electrical field (Δ*C* + *E*) and concentration gradient field coupled with light-induced heat field and electrical field (Δ*C* + *hv* + *E*). The transport system was employed to selectively extract Li^+^ from a lithium-containing leaching solution, which is obtained from a battery recycling factory and generated during the dismantling and recycling of spent LIBs. **b** Polyhedral representation of the rhombohedral crystal structure of LAGP. Dark grey: Li; cornflower blue: Ge/Al, light green: P, light coral: O. **c** BVEL maps with isosurfaces (yellow) drawn at the 1.4 eV level above *E*_min_. Li-ion migration channels of rhombohedral LAGP are obtained from crystal structure analysis and bond valence energy landscape (BVEL) method. The potential diffusion pathway is illustrated by a continuous yellow band area, and visualization of the positions of three Li-ions, Li(1): light coral sphere, Li(2): dark grey sphere, and Li(3): light green sphere

Here, we demonstrate the MSITS based on heterogeneous nanofluidic membrane Li_1.5_Al_0.5_Ge_1.5_P_3_O_12_ (LAGP)/(multi-walled carbon nanotubes/cellulose nanofiber) (MWCNTs/CNF), assisted by multi-external fields (i.e., light-induced heat, electrical and concentration gradient fields) (Fig. 1a). Heterogeneous nanofluidic membrane retains the intrinsic structure and properties of LAGP, with three-dimensional lithium channels, contributing to high ion selectivity and low mass transfer resistance. In addition, MWCNTs/CNF functions as a photothermal layer due to its excellent photothermal conversion effect, which contributes to improving the ion transmembrane migration by increasing ionic mobility of the ion-enrichment zone at the membrane interface and suppressing the ICP effect. The Li^+^ flux of the MSITS can be up to 367.4 mmol m^−2^ h^−1^ as extracting Li from the spent LIBs leaching solutions, an approximately 1300% increase compared with the system driven by concentration gradient field. Furthermore, the proposed transport system exhibits ultrahigh Li^+^ selectivity with factors of 216,412, 142,043, 51,843, 23,276, and 3907, for Li^+^/Co^2+^, Li^+^/Mn^2+^, Li^+^/Cu^2+^, Li^+^/Ni^2+^, and Li^+^/Na^+^, respectively, outperforming the previous reports of Li-ion sieving systems. The significant performance improvement shows the practical potential of our strategy, indicating the imperative of multi-field-coupled strategy in membrane separation resource extraction, and it also provides a technical reference for the novel Li^+^ enrichment technology based on nanofluidic membrane separation system.

## Experimental and Calculation

### Fabrication of the Heterogeneous Membrane

The heterogeneous membrane was fabricated by the dip-coating method. Firstly, the CNF gel was dissolved in deionized water to obtain a uniform dispersion (5 mg mL^−1^) after ultrasonication. And then 5 mL MWCNTs-COOH suspension (12 mg mL^−1^) was added to 1 mL CNF dispersion to form a mixed solution, which was sonicated for 10 min and stirred for 1 h to form a uniform suspension. The MWCNTs/CNF suspension was drop-cast on a LAGP pellet and dried in a 60 °C oven. The evaporation of solvent will result in a uniform coating of the MWCNTs/CNF layer, called the photothermal layer, thereby achieving the preparation of a heterogeneous membrane.

### Characterizations

The morphology of heterogeneous membranes was observed using scanning electron microscopy (SEM, Hitachi S-4800). X-ray diffraction (XRD) pattern was carried out on a Bruker D8 Focus Powder diffractometer with a Cu Kα radiation source (*λ* = 0.154 nm). Fourier transform infrared (FTIR) spectra were performed on an Excalibur 3100 Fourier transform infrared spectrometer (Varian, USA). Raman spectra were measured by an inVia-Reflex microscope spectrometer (Renishaw, UK) with the radiation of a 532-nm laser. X-ray photoelectron spectroscopy (XPS) spectra were collected on an Al Kα source (ESCALAB 250Xi). The zeta potential of MWCNT/CNF was evaluated using a Zetasizer Nano (ZS90, Malvern Instruments Ltd., Malvern, England). The contact angles of the membranes were measured at room temperature using an OCA25 contact angle measuring instrument (Dataphysics, Germany).

### Photothermal Conversion Property Measurements

Membranes were used for the photothermal conversion performance measurement. Light with different wavelengths was applied at a light intensity of ~ 250 mW cm^−2^. The temperature of the heterogeneous membrane was monitored every 5 s using a Fluke thermal imaging camera, and the measurement accuracy of the camera is ± 2 °C. (Ti450; Everett, Washington, USA).

### Electrical Measurements

The electrical measurements were conducted using Keithley 6430 (Keithley Instruments, Cleveland, OH). For the ionic transport properties of current–voltage curve, the electrolytes in the two cells are the same. For energy conversion properties, the low-concentration electrolyte faced the MWCNTs/CNF layer, while the high-concentration electrolyte faced the LAGP layer. Ag/AgCl electrodes were used to collect the current and voltage signals, with the anode positioned on the high-concentration electrolyte side. With respect to light density-dependent measurements, the light density can be controlled by adjusting the instrument output light intensity density.

### Theoretical Calculations

All the calculations are performed in the framework of the density functional theory (DFT) with the projector augmented plane-wave method, as implemented in the Vienna ab initio simulation package. The generalized gradient approximation proposed by Perdew, Burke, and Ernzerhof is selected for the exchange–correlation potential. The cutoff energy for the plane wave is set to 450 eV. The energy criterion is set to 10^−4^ eV in the iterative solution of the Kohn–Sham equation. The Brillouin zone integration is performed using a 2 × 2 × 1 k-mesh. All the structures are relaxed until the residual forces on the atoms have declined to less than 0.02 eV Å^−1^.

The bond valence energy landscape (BVEL) analysis [38–40], based on the theory that transport pathways can be evaluated by calculating the accessible sites for mobile ions, has been used as an effective ionic migration pathways simulation tool. Our BVEL methods take 10 Å as the cutoff radius and 0.1 Å as the grid resolution to obtain the mobile ions migration energy barrier by defining the Morse and Coulombic potentials as the following formulas:

1 $$E\left( {{\text{Li}}} \right)_{{{\text{morse}}}} = D_{0} \left\{ {\left( {\exp \left[ {\alpha \left( {R_{\min } - R} \right)} \right] - 1} \right)^{2} - 1} \right\}$$

2 $$E\left( {{\text{Li}} - A} \right)_{{{\text{coulomb}}}} = \frac{{q_{{{\text{Li}}}} q_{A} }}{{R_{{{\text{Li}} - A}} }}{\text{erfc}}\left( {\frac{{R_{{{\text{Li}} - A}} }}{{\rho_{{{\text{Li}} - A}} }}} \right)$$

where *D*_0_, α, *R*_min_ are morse potential parameters determined from a large amount of stable compounds and *q* and *R* refer to effective charges of atoms and bond distance between atoms, respectively.

To complete the analysis of ionic migration, the quasi-empirical BVEL methods with high calculation efficiency are employed to obtain the basic information on possible ionic migration channels for LAGP. By adding the morse potential energy of mobile ions and the related anions and the coulomb repulsion potential of mobile ions and cations to the basic approximate methods, BVEL has been performed to calculate the ionic migration pathways for LAGP.

## Results and Discussion

### Strategy and Characterizations

As shown in Fig. S1, the heterogeneous LAGP/(MWCNTs/CNF) membrane, prepared by drop coating method, is assembled into a designed transfer system which is divided into feed and receiving chambers by the membrane. The heterogeneous nanofluidic membrane consists of LAGP and MWCNTs/CNF layer, wherein the thicknesses of corresponding portions are approximately 260 and 1.2 μm, respectively. The specially designed structure results in rapid Li^+^ transport properties and external field responsivity. Transport systems under the diverse external field to promote ion transport across nanofluidic membranes are illustrated in Fig. 1a. The behavior of transmembrane transport under different driving forces (Δ*C*, Δ*C* + *hv*, Δ*C* + *E*, and Δ*C* + *hv* + *E*) was investigated and analyzed detailedly in the subsequent sections. The local magnification in Fig. S1 indicates that the LAGP demonstrated a typical continuous dense structure, serving as a selective layer that controls Li-ion selective transport across the membrane. LAGP was a rhombohedral lithium superionic conductor [41–43], whose basic structure consists of PO_4_ tetrahedra, GeO_6_/AlO_6_ octahedra and LiO_6_ units in trigonal antiprismatic coordination (Fig. 1b). The Li (denoted as Li(1) in the following), Ge/Al, P and O ions occupied the crystallographic Wyckoff 6b, 12c, 18e, and 36f sites, respectively. Heterovalent cation substitution introduced additional Li, located in Li(2) and Li(3) sites, in each formulation unit for charge compensation, enabling a further increase in ionic conductivity by increasing carrier concentration [44].

Understanding the three-dimensional diffusion process of Li^+^ in LAGP has obvious implications for revealing the ionic transmembrane transport process [45, 46]. As shown in Fig. 1c, we conducted an in-depth analysis of Li-ion diffusion channels in rhombohedral LAGP by integrating crystal structure analysis and bond-valence energy landscape (BVEL) analysis. Based on the theory that transport pathways could be evaluated by calculating the accessible sites for mobile ions, the BVEL method has served as an effective ionic migration pathways simulation tool [47, 48]. According to Fig. 1c, the topology of the ionic-conduction pathways is consistent with BVEL maps of Li_1.5_Al_0.5_Ge_1.5_(PO_4_)_3_, where the isosurfaces (yellow) illustrated that conduction occurs along the continuous zigzag channel containing sites associated with Li. The transport mechanism in LAGP materials was considered a concerted migration mechanism, which involved the simultaneous correlated motion of several neighbouring atoms [49]. Aside from the configuration, the coulomb interaction between migrating and neighbouring mobile ions was critical to orchestrating concerted migration. Migration barriers for correlated motions of several mobile ions tended to be lower than those for uncorrelated single ion hopping. Additionally, the MWCNTs/CNF layer acted as the photothermal layer to enhance ion transport [50]. By illuminating the MWCNTs/CNF layer, its excellent photothermal conversion property enabled the membrane surface to be heated up, which contributed to the increase of ion thermophoresis mobility and accelerated the diffusion of ions at the membrane interface. The ICP phenomenon was reflected by the formation of an ion-depletion zone at the surface facing the concentrated solution, and an ion-enrichment zone comprising the target ions transported across the membrane at its opposite side. Therefore, the diffusion boundary layer adjacent to the membrane induced by ICP weakened the driving force for preferential ion transport across the membrane, thereby inhibiting the practical application of numerous membrane separation processes. While the above weakening effect to driving force was effectively released by suppressing ICP through light-induced heat-promoting ion transport at the membrane interface. Overall, the functional nanofluidic membrane could integrate with a coupled multiple-driven force to achieve the efficient recovery of specific ions.

### Evaluation of Electrochemical Properties

**Fig. 2:**
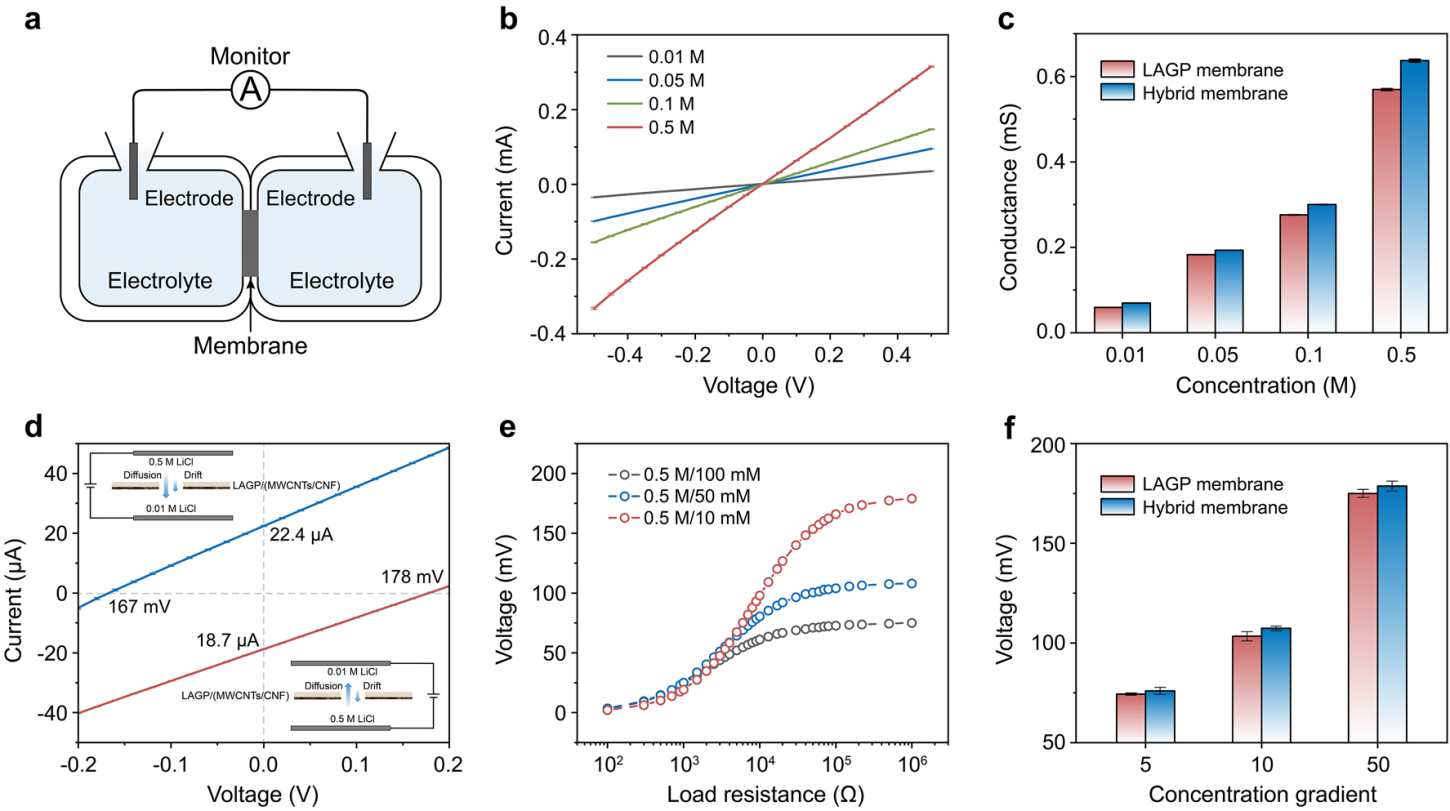
Transmembrane ionic transport. **a** Schematic illustration of the experimental setup to measure transmembrane ionic transport. **b** *I*–*V* curves of HM measured in LiCl electrolyte with different concentration ranging from 0.01 to 0.5 M. **c** Conductance measurement of the LM and HM in LiCl electrolyte. **d** *I-V* curves of the HM at a 50-fold gradient under forward and backward LiCl concentration gradient. **e** Plot of voltage as a function of load resistance in the transport system equipped with HM. The harvested energy under a concentration gradient can be transferred to supply an external resistance. Under 5, 10, and 50-fold concentration gradients, the measured voltages all gradually increase with increasing load resistance. **f** The transmembrane potential value of the LM and HM

The ion transport properties of the LAGP membrane (LM) and heterogeneous membrane (HM) were investigated with a homemade symmetric double electrochemical cell, as shown in Fig. 2a. Ion current–voltage (*I*–*V*) curves were conducted in LiCl solutions at concentrations ranging from 0.01 to 0.5 M, all exhibiting linear ohmic behavior (Figs. 2b and S2). The ionic conductance was derived from the corresponding *I*–*V* curves (Fig. 2c). Notably, the ionic conductance of the HM with an additional MWCNTs/CNF photothermal layer was slightly higher than that of the bare LM, indicating that the addition of the photothermal layer positively promoted ion transport even without light illumination. The enhancement was realized via surface negative charge and wettability [51, 52]. In detail, the water contact angle of the HM (photothermal layer side) was reduced to 59° from 103° for that of bare LM (Fig. S3), owing to the presence of hydrophilic groups (carboxyl, hydroxyl) within the MWCNTs/CNF photothermal layer, as confirmed by XPS analysis and FTIR spectrum analysis (Figs. S4–S5) [53, 54]. In addition, MWCNTs/CNF was negatively charged in a neutral aqueous solution with a zeta potential of ~ − 33 mV (Fig. S6).

Osmotic energy is well known as a sustainable and clean energy source for the future [55, 56]. When constructing an ion transport system utilizing a functional membrane (i.e., ion selective permeation membranes), the specific ions could be selectively transported directionally across the membrane driven by the concentration gradient field, enabling the enrichment and recovery of the target ions. It is worth noting that part of the Gibbs free energy contained in the concentration gradient could also be converted to an external circuit. To evaluate the osmotic energy-driven ion transport properties, LiCl solutions (0.5/0.01 M) were applied across the HM. Two configurations could be arranged for the concentration gradient. The transmembrane ion currents were recorded under a concentration gradient from the LAGP side fixed at 0.5 M concentration to the MWCNTs/CNF side fixed at 0.01 M concentration (blue curve in Fig. 2d) and under the reverse concentration gradient (red curve in Fig. 2d). The short-circuit current (*I*_sc_) and open-circuit voltage (*V*_oc_) were 22.4 μA and 167 mV, respectively, when the low-concentration solution was placed in the MWCNTs/CNT side. Under the reverse concentration gradient with low-concentration solution placed in the LAGP side, the corresponding *I*_sc_ and *V*_oc_ are 18.7 μA and 178 mV, and the calculated internal resistance was increased by ~ 28%, which severely suppresses the ion transmembrane transport performance. The former concentration gradient configuration had a smaller internal resistance and a larger* I*_sc_ of 22.4 μA, as shown in Fig. 2d, which was considered a favourable direction. Regarding ion transport driven by concentration gradient, the driving force could be evaluated by voltage (*U* = *I* × *R*_L_, *R*_L_ is the load resistance of the external circuit). The voltage increased as loading resistance, eventually reaching a plateau with the internal resistance was much smaller than the load resistance, which approximately corresponds to the output voltage based on the osmotic energy driving ion transport across the membrane. As the concentration gradient gradually increased from 5 to 50-fold, the output voltage rose from 75 to 175 mV (Fig. 2e). The corresponding current density and power density are shown in Figs. S7–S8, of which the power density also increases as raising concentration gradient. The opposite trend of current density, which decreases with an increasing concentration gradient, is caused by the low conductivity of the corresponding low-concentration solution, indicating that it is advisable to add a supporting electrolyte to the receiving solution in later separation tests. The output voltage of the HM is slightly higher than that of the LM (Figs. 2f and S9), indicating that the ions within the transport system assembled with the HM are subject to a heightened driving force for transmembrane transport at the same concentration gradient, which is more conducive to directional transport ions.

### Photothermal Effect of the HM

Carbon-based nanomaterials (such as MWCNTs) emerged as preferred candidates for photothermal materials because of their low cost, ease of processing, and excellent photothermal activity, comprising both light absorption and thermal conductivities. Light, carrying photonic energy, which could be absorbed when light interacts with the MWCNTs/CNF photothermal layer, leading to the photoexcitation of electrons. The energy of the excited electrons was transferred to the lattice of *sp*^2^ or *sp*^3^ through electron–phonon coupling (phonons account for the majority) thereby generating heat. Raman spectrum verified the coexistence of *sp*^2^ and *sp*^3^ carbon atoms in the MWCNTs/CNF layer (Fig. S10) [57, 58]. Moreover, the MWCNTs within the photothermal layer exhibit broadband adsorption owing to the wide distribution of diverse chirality. Efficient photothermal conversion allowed for intense heat accumulation induced by excellent light absorption capability, contributing to temperature redistribution.

**Fig. 3:**
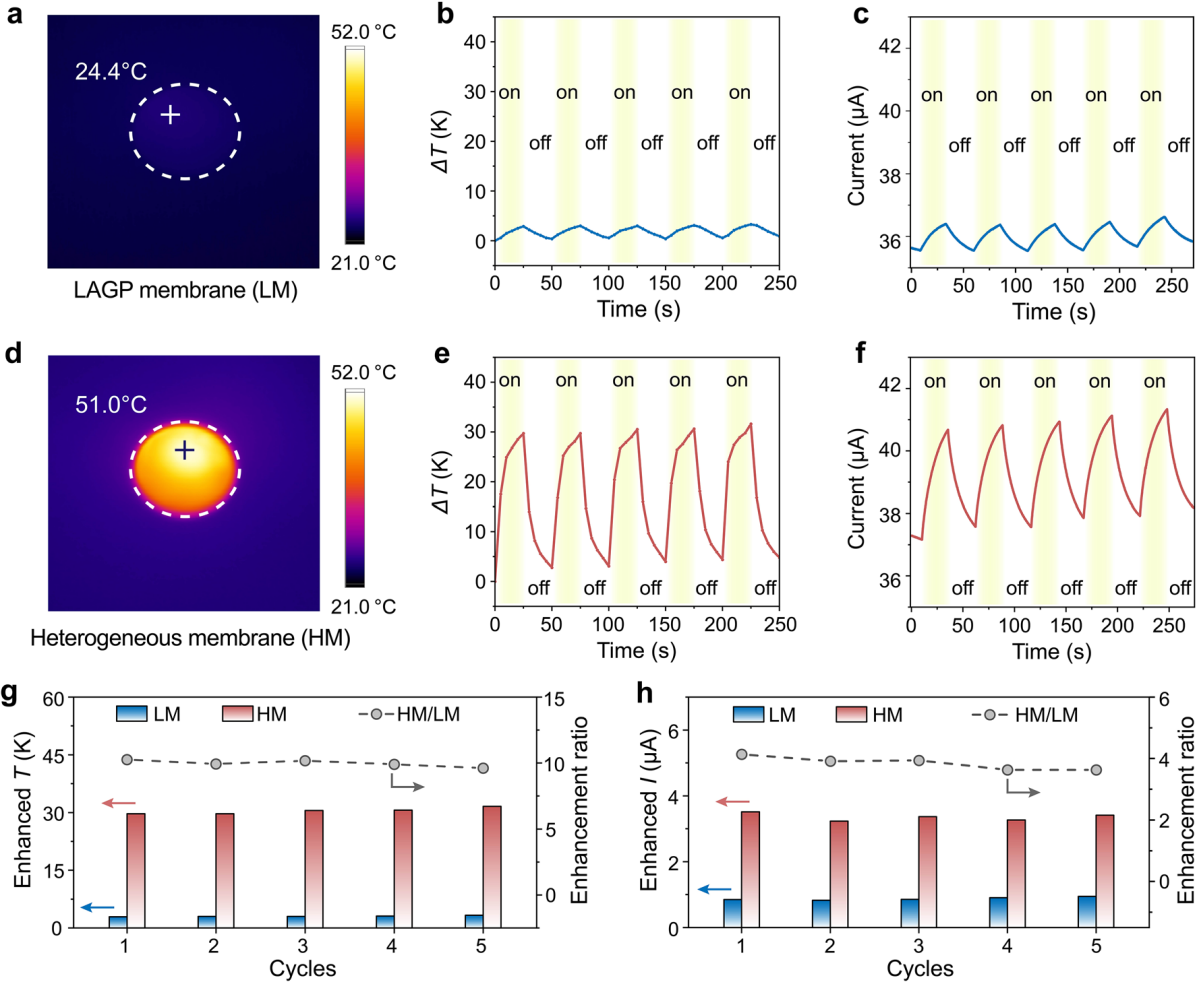
The photothermal effect of the LM and HM. **a** IR camera image of the LM under light irradiation. Light-induced **b** temperature changes and **c** ionic current changes of the LM under alternating illumination. **d** IR camera image of the HM under light irradiation. Light-induced **e** temperature changes and **f** ionic current changes of the HM under alternating illumination. **g** Comparison of the enhanced temperature (*T*) of the LM and HM after a certain time (25 s) of light irradiation. **h** Comparison of the enhanced current (*I*) of the LM and HM after a certain time (25 s) of light irradiation

The temperature distributions measured by infrared thermography and the corresponding ion currents with and without illumination were employed to evaluate the photothermal behavior of the nanofluidic membranes under different conditions. Figure 3a demonstrates the infrared (IR) image of the LM upon light irradiation. Light induced a slight increase in surface temperature (Fig. 3b) and corresponding current (Fig. 3c) for LM. However, the surface temperature of the HM was significantly elevated to 51 °C under light irradiation condition (Fig. 3d), which ascribed to the excellent photothermal conversion and light absorption capabilities of the photothermal layer of the HM. In the illumination cycling test, the temperature of the HM increased rapidly (~ 30 °C, Fig. 3e), and the corresponding ion current increased sharply to 41.5 μA (Fig. 3f), exhibiting light-induced heat promoted ion migration. When the illumination was stopped, the temperature and current values decreased and returned to the initial state, demonstrating excellent cycling stability. Under unilateral illumination, the temperature of the photothermal layer increased under illumination, and the corresponding electrolyte at the membrane interface in contact with the photothermal layer is also heated. Benefiting from the temperature redistribution caused by the photothermal conversion, the ion thermophoresis migration is enhanced. And the ICP effect was effectively suppressed by the impairing ion-enrichment zone through accelerating ionic diffusion from the membrane interface to the bulk solution, which improved the ion transport performance and enhance the recovery efficiency of Li-ions. In addition, the LM also exhibited a minor photothermal effect, while significantly less than that of the HM with a photothermal layer (Fig. 3g, h). The enhancement ratios of temperature (*T*) and current (*I*) are approximately 10 and 3.8. Thus, the light-induced heat field could successfully accelerate the directional transport of specific ions through thermal gradients, allowing for the efficient extraction of ions from resources.

### Multi-field-coupled Synergistic Ion Transport

**Fig. 4:**
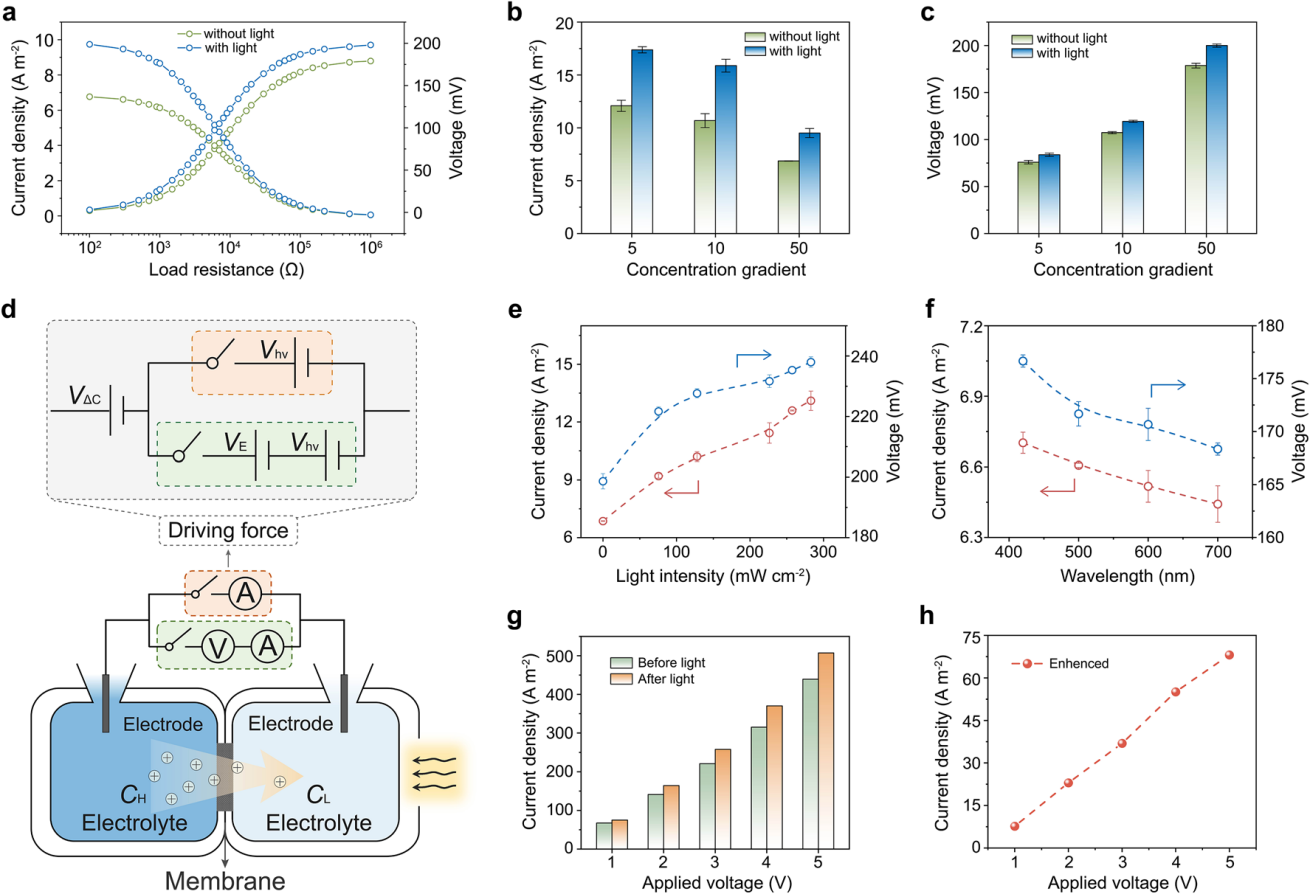
MSITS transmembrane ionic transport. **a** The plot of current density and voltage as a function of load resistance under the condition of with (blue line) and without (green line) light irradiation. Comparison of** b** the current density and **c** voltage of the HM under various concentration gradients with and without light irradiation. **d** Schematic of the MSITS including concentration gradient field coupled with light-induced heat field (Δ*C* + *hv*) and concentration gradient field coupled with light-induced heat field and electrical field (Δ*C* + *hv* + *E*). Inset: equivalent circuit of the schematic, *V*_Δ*C*_ represents the ion-driven force derived from the concentration gradient, *V*_*E*_ represents the applied voltage, and *V*_*hv*_ represents the additional ion-driven force under light derived from photothermal conversion. **e** The relationship between the voltage and current density of the HM at a 50-fold gradient with the light intensity. **f** The relationship between the voltage and current density of HM at a 50-fold gradient with the wavelength. **g** The ion current of the transport system applied with concentration gradient and various electric voltages before light irradiation and after light irradiation for a certain time of 25 s. **h** The enhanced current of the HM under light irradiation

Furthermore, the dual-field and multi-field coupled transport systems were investigated in detail. When light irradiated, photothermal conversion renders the enhancement of temperature upon the HM surface. According to Einstein relation, an increase in temperature would lead to an increase in ion migration, allowing for a significant reduction in ICP effects that impair ion transport. Figure 4a demonstrates the curves of current density and voltage as the function of load resistance in the circuit, in which the corresponding curves under light irradiation were higher than that without light. The current densities and voltages of the HM in different concentration gradients were significantly higher with light than without light (Fig. 4b, c). Owing to the temperature redistribution induced by the photothermal effect, the ions were driven to accelerate across the HM, prompting higher current density and voltage values with light irradiation than without it (Figs. S11–S14). Figure 4d illustrates the schematic of the dual-field and multi-field coupled transport system including concentration gradient field coupled with light-induced heat field (Δ*C* + *hv*) and concentration gradient field coupled with light-induced heat field and electrical field (Δ*C* + *hv* + *E*). Notably, the voltage increased from 177 to 220 mV by increasing the light intensity, and similarly, the current density increased from 7 to 13 A cm^−2^ (Figs. 4e and S15). Moreover, the wavelength dependence of the light-promoted ion transport process was investigated, which attributes to the variation in light absorption intensity at specific wavelengths, as evaluated by the ultraviolet–visible (UV–Vis) spectroscopy (Fig. S16). Since the light absorption intensity of the photothermal layer declines with the increase of wavelength, the ion current and voltage also decreased with the increase of wavelength. The voltage was reduced from 177 to 168 mV, and the current density drops from 7.2 to 6.7 A cm^−2^, as shown in Figs. 4f and S17. It was indicated that the light absorption capacity of the photothermal layer has a significant effect on light-enhanced ion transport.

In addition to the concentration gradient field coupled with the light-induced heat field to accelerate ion transmembrane transport, the voltage-driven force could be introduced into our transport system as an ideal driving force owing to its sustainability and tunability. Applying a forward voltage to the transport system equipped with a concentration gradient, the ionic current increased with raising applied voltage following Ohm's law (Fig. 4g). Applying light-induced heat field to the above system, the ion current was further enhanced, and the enhancement of which increases with increasing value of applied voltage, which could be attributed to multiple external fields coupling positively promoting ion transport across the membrane (Fig. 4h). The strategy proved to be functional in accelerating ion transmembrane transport.

### Lithium Recovery from Spent LIBs

**Fig. 5:**
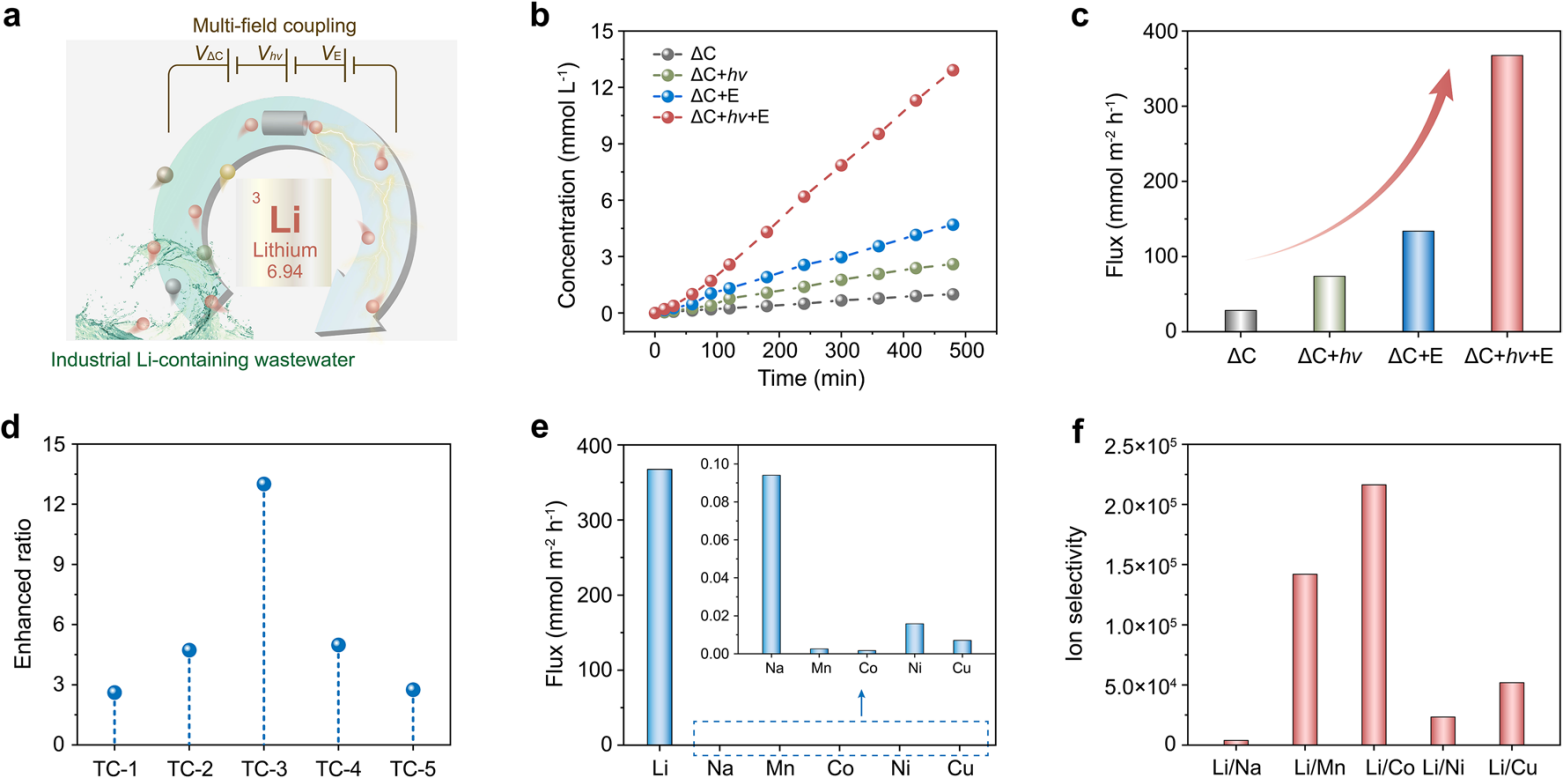
Lithium recovery from spent LIBs. **a** Schematic of the MSITS used for extracting lithium from spent LIBs. **b** Variation of lithium-ion concentration in the receiving solution with operating time under different coupled transport systems. The lithium-ion concentration is much higher in the MSITS (Δ*C* + *hv* + *E*) than in other external field-applied conditions. **c** Ion fluxes of the transport system under different external field application conditions. **d** The enhanced ratios of flux for two different external field (TC) application conditions. TC-1 to TC-5 corresponding to the (Δ*C* + *hv*)/Δ*C*, (Δ*C* + *E*)/Δ*C*, (Δ*C* + *hv* + *E*)/Δ*C*, (Δ*C* + *hv* + *E*)/(Δ*C* + *hv*), and (Δ*C* + *hv* + *E*)/(Δ*C* + *E*), respectively. **e** Ion fluxes of the MSITS for the extraction of Li^+^ from lithium-containing leachate generated during the dismantling and recycling of spent LIBs. Inset: ion fluxes of other coexisting ions, which are extremely small compared to lithium, are barely visible in the same coordinate range. **f** Ion selectivity of Li ions with other coexisting metal ions

The MSITS was systematically investigated to effectively extract lithium from industrial Li-containing wastewater, which was the leaching solution obtained from a battery recycling factory and generated during the dismantling and recycling of spent LIBs (Fig. 5a). The experimental setup and ion concentration were described detailly in electrical measurements part (Fig. S18, Table S1). External fields included concentration gradient field (Δ*C*), concentration gradient field coupled with light-induced heat field (Δ*C* + *hv*), concentration gradient field coupled with electrical field (Δ*C* + *E*), and concentration gradient field coupled with light-induced heat field and electrical field (Δ*C* + *hv* + *E*). According to Fig. 5b, the Li^+^ concentration in the receiving compartment increases linearly over time, and the increase rate was dependent upon the external field coupling condition. A multi-field-coupled synergistic driving condition contributed to a significantly higher ion concentration than a single or dual external field driving condition. The excellent photothermal conversion property enabled the temperature enhancement of the membrane interface as light irradiates the photothermal layer of the HM, facilitating an increase in ion thermophoresis mobility and a reduction in ICP. With the introduction of electrical field into the transport system, ion transmembrane transport was accelerated, accompanied by an apparent increase in Li^+^ concentration in the recovery compartment. While the electrical field may induce more severe ICP around the membrane interface, at this time the temperature gradient induced by photothermal conversion effectively restricted the expansion of the ICP zone. At the condition of applying multi-external field (Δ*C* + *hv* + *E*), the Li^+^ flux is 367.4 mmol m^−2^ h^−1^, which was significantly higher than that of others and even higher than the summation of the other conditions, indicating that the synergistic effect of multiple external fields improved ion transmembrane transport and thus contributed to ions enrichment in the receiving compartment (Fig. 5c). We investigated the enhanced ratios of flux for different external field application conditions, as shown in Fig. 5d, for two different external fields TC-1 to TC-5 corresponding to (Δ*C* + *hv*)/Δ*C*, (Δ*C* + *E*)/Δ*C*, (Δ*C* + *hv* + *E*)/Δ*C*, (Δ*C* + *hv* + *E*)/(Δ*C* + *hv*), and (Δ*C* + *hv* + *E*)/(Δ*C* + *E*), respectively. Li^+^ flux in multiple external fields (ΔC + *hv* + E) was 13-fold higher than that in an individual concentration gradient field (Δ*C*), indicating that a significant enhancement of ion transport could be achieved by modulating the external fields applied to the system. For (Δ*C* + *hv*)/Δ*C* and (Δ*C* + *hv* + *E*)/(Δ*C* + *E*), the introduction of the light-induced heat field increases the ion flux to 2.61 and 2.75-fold of the original external field, respectively. Also, the electrical field was observed to exert a further driving force for ion transmembrane transport, leading to flux ratios of 4.7 and 5-fold for (Δ*C* + *E*)/Δ*C* and (Δ*C* + *hv* + *E*)/(Δ*C* + *hv*) as well. The MSITS exhibits high selectivity for other ions when used to extract Li-ions from spent LIBs, which could be attributed to the intrinsic crystal structure of LAGP. The lithium-containing leaching solution was selected as the feed solution mainly containing Li^+^ and other coexisting ions such as Na^+^, Ni^2+^, Mn^2+^, Cu^2+^, and Co^2+^. Under a MSITS (Δ*C* + *hv* + *E*), the Li^+^ flux (367.4 mmol m^−2^ h^−1^) is much higher than the Na^+^, Mn^2+^, Co^2+^, Ni^2+^, and Cu^2+^ flux (0.094, 2.59 × 10^−3^, 1.7 × 10^−3^, 0.015, and 7.09 × 10^−3^ mmol m^−2^ h^−1^, respectively), as shown in Fig. 5e. The ion selectivity (ratio of Li^+^ flux to other metal ion flux during the test process), measured by ICP-MS, is shown in Fig. 5f. The MSITS (Δ*C* + *hv* + *E*) displayed selectivity for Li^+^/Na^+^ of 3907, Li^+^/Mn^2+^ of 142,043, Li^+^/Co^2+^ of 216,412, Li^+^/Ni^2+^ of 23,276, and Li^+^/Cu^2+^ of 51,843. The Li^+^ was preferentially transport across heterogeneous nanofluidic, and other cations were intercepted in the feed solution, allowing the selective extraction of lithium resources and the recycling of lithium resources within spent LIBs. Although recently reported nanofluidic membranes exhibited promising Li^+^ selectivity, as shown in Fig. S19 and Table S2, the selectivity was relatively low for most porous membrane materials, including MOF-based membranes, polymer membranes, 2D-based membranes, and COF-based membranes. Our work demonstrated a significantly higher lithium-ion selectivity than other membranes mentioned above and provide a methodology for the design of a Li extraction device. Considering the widespread use of LIBs in contemporary energy applications, optimizing this highly effective lithium extraction system is expected to ease the looming shortage of lithium resources.

## Conclusions

In summary, we demonstrated a MSITS based on an elaborate heterogeneous nanofluidic as a promising platform for Li extraction. LAGP provided Li-ion selectivity for its intrinsic crystal structure, and the MWCNTs endowed the heterogeneous nanofluidic with an excellent photothermal conversion property, for amplifying the thermophoretic mobility of ions and alleviating the ICP effect. We adopted a multi-field-coupled synergistic ion transport strategy for lithium recovery from spent LIBs. The MSITS demonstrates a significantly higher Li^+^ ion flux of 367.4 mmol m^−2^ h^−1^, which was improved to 1300% of the flux at a single concentration gradient field. Benefiting from the efficient coordination of multi-external fields and heterogeneous nanofluidic membrane, the proposed transport system exhibited ultrahigh Li-ion selectivity, achieving Li^+^/Co^2+^, Li^+^/Mn^2+^, Li^+^/Cu^2+^, Li^+^/Ni^2+^, and Li^+^/Na^+^ selectivity with ratios of 216,412, 142,043, 51,843, 23,276, and 3907, respectively, outperforming reported Li-ion sieving systems. Our work presented an ideal strategy for designing an extraction system by integrating multiple external fields to ensure efficient resource recovery. This may further inspire the exploration of the next-generation advanced transport system with multiple intelligences to improve recovery, seawater desalination, and osmotic energy conversion.

### Supplementary Information

Below is the link to the electronic supplementary material.

Supplementary file1 (PDF 1712 KB)
