# Supplementary material for: Engineering Multi-field-coupled Synergistic Ion Transport System Based on the Heterogeneous Nanofluidic Membrane for High-Efficient Lithium Extraction
Source: Nanomicro Lett. 2023 May 20;15:130. doi: 10.1007/s40820-023-01106-5 (PMC10200000; doi:10.1007/s40820-023-01106-5)
Supplement: Supplementary file 1 — Supplementary file1 (PDF 1712 KB) [file 40820_2023_1106_MOESM1_ESM.pdf]

Supporting Information for

Engineering Multi-Field-Coupled Synergistic Ion Transport System Based on the Heterogeneous Nanofluidic Membrane for High-Efficient Lithium Extraction

Lin Fu[1, 2], Yuhao Hu[1, 2], Xiangbin Lin[1, 2], Qingchen Wang[1, 2], Linsen Yang[1, 2], Weiwen Xin[1, 2], Shengyang Zhou[1, 2], Yongchao Qian[1], Xiang-Yu Kong[1, 2, *], Lei Jiang[1, 2], and Liping Wen[1, 2, 3, *]

[1]CAS Key Laboratory of Bio-inspired Materials and Interfacial Science, Technical Institute of Physics and Chemistry, Chinese Academy of Sciences, Beijing 100190, P. R. China

[2]School of Future Technology, University of Chinese Academy of Sciences, Beijing 100049, P. R. China

[3]Qingdao Institute of Bioenergy and Bioprocess Technology, Chinese Academy of Sciences, Qingdao 266101, P. R. China

*Corresponding authors. E-mail: wen@mail.ipc.ac.cn (Liping Wen), kongxiangyu@mail.ipc.ac.cn (Xiang-Yu Kong)

S1 Supplemental Notes

S1.1 Materials

Cellulose nanofiber (CNF) gel (1.20 wt%) was purchased from Tianjin Woodelfbio Cellulose Co., Ltd. Carbonylated multiwalled carbon nanotube (MWCNTs-COOH, 20 wt%) was provided by XFNANO, China. $Li_{1.5}Al_{0.5}Ge_{1.5}(PO_3)_4$ (LAGP) pellets were obtained from GanFeng Lithium Co., Ltd. Lithium chloride (LiCl, 99.9 wt%) was purchased from Sigma-Aldrich. Potassium ferrocyanide ($K_4Fe(CN)_6$, 99 wt%), potassium ferricyanide ($K_3Fe(CN)_6$, 99 wt%), and ammonium sulfate ($(NH_4)_2SO_4$, 99 wt%) were purchased from Aladdin. Deionized (DI) water (18.25 MΩ·cm) was used in all the experiments. All of the chemicals were of analytical grade.

S1.2 Electrical Measurements

The ionic transport and energy conversion properties were studied by measuring the zero-volt ionic current and corresponding circuit voltage through the heterogeneous membrane or LAGP membrane with and without light illumination. The membrane was mounted between two chambers of the homemade cells with silicone O-rings, which are filled with electrolytes with a certain concentration and type. One of the electrolyte cells with a light window, through which the photothermal layer could be illuminated.

Regarding ion transport driven by concentration gradient, the internal resistance was equal to the load resistance when the power density is maximum. In addition, the load resistance (1,000 kΩ) was more than two orders of magnitude higher than the internal resistance of various concentration gradient conditions, indicating the partial voltage of internal resistance could be neglected. Thus, the driving force derived from concentration gradient was simplified to the follows:

$$U = I \times R_L \qquad \text{(S1)}$$

I is the current of the transport system, R_L is the load resistance, and U is the partial voltage of the R_L in the circuit, which can be considered as the driving force derived from the concentration gradient as the load resistance is much larger than internal resistance.

S1.3 Lithium Recovery from Spent LIBs

The multi-external field coupled transport system setup for lithium recovery from spent LIBs was classified into three compartments and designated as electrode compartments (cathode and anode), feed compartment, and recovery compartment. Considering the poor light stability and the excessive consumption of Ag/AgCl electrodes during long-term operation, titanium electrodes with RuO_2-IrO_2 coatings (Suzhou Borui Industrial Material Science & Technology Co., Ltd. China) were selected for their superior thermal stability and corrosion resistance and placed in the electrode compartments as anode and cathode electrodes. A heterogeneous nanofluidic membrane with an effective testing area of 153 mm^2 was placed between the feed and recovery compartments, with the photothermal layer facing the recovery compartment and the LAGP layer facing the feed compartment. Anion exchange membranes (AEM, Selemion™ AAV) are placed between the electrode solution and feed solution or recovery solution, allowing selective anion transport across the membrane to maintain electroneutrality. The electrochemical measurements were carried out on a Keithley 6430 semiconductor picoammeter (Keithley Instruments, Cleveland, OH) by applying a voltage of 5 V. The light intensity applied to the system is ~250 mW cm^{-2}. The electrode solution is a mixture of 0.1 mol L^{-1} $K_4Fe(CN)_6$, 0.1 mol L^{-1} $K_3Fe(CN)_6$, and 0.5 mol L^{-1} $(NH_4)_2SO_4$. A solution of 0.05 mol L^{-1} $(NH_4)_2SO_4$ was used as a receiving solution. The feed compartment was a lithium-containing leaching solution (Table S1), which was obtained from a battery recycling factory and generated during the dismantling and recycling of spent LIBs (mainly ternary LIBs). The feed and receiving compartments are equal to 35 mL. Peristaltic pumps with a flow rate of 20 mL min^{-1} were employed to avoid concentration polarization. The metal ions concentrations in the recovery compartment were tested by ICP-MS (PerkinElmer 1000G) to calculate the ions flux and ion selectivity using the following expression:

$$J = \frac{(C_f - C_i) \cdot V}{A_m \cdot t} \qquad \text{(S2)}$$

J is the metal ion flux (mmol m^{-2} h^{-1}), C_f and C_i are the metal ion concentration in the recovery compartment at the final time and initial moment, V is the volume of recovery solution, A_m is the effective area of the heterogeneous membrane, and t is the time corresponding to ion transmembrane transport:

$$\eta = \frac{J_{Li}}{J_M} \qquad \text{(S3)}$$

η is ion selectivity, J_{Li} is the ion flux of Li ions, and J_M is the ion flux of other coexisting metal ions.

S2 Supplementary Figures

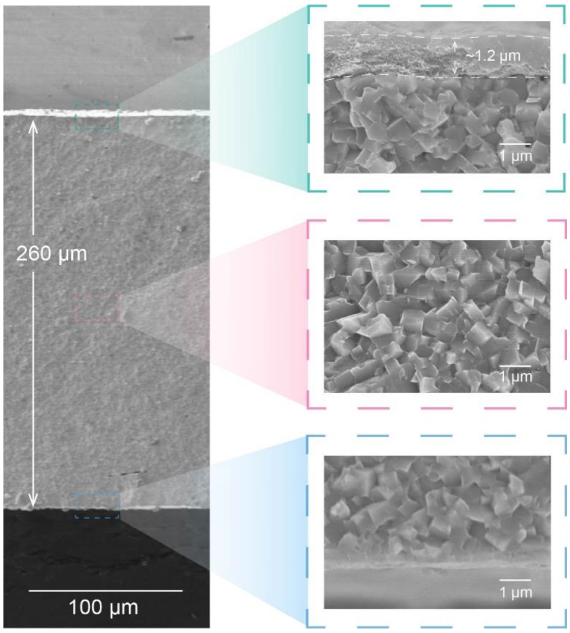

Fig. S1 SEM image of the heterogeneous membrane, demonstrating the thickness is about 260 μm. The local magnification indicates that the thickness of the photothermal layer is 1.2 μm. In addition, the LAGP demonstrates a typical continuous dense structure, which ensures the ion selectivity.

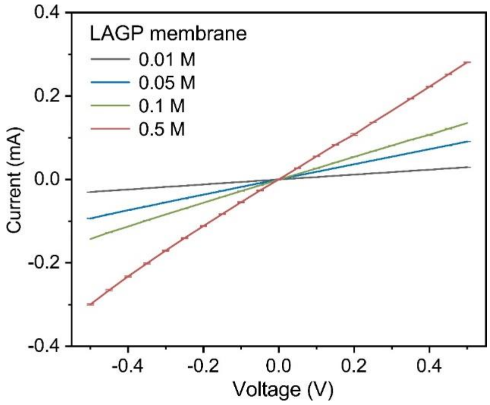

Fig. S2 *I-V* curves of LAGP membrane measured in LiCl electrolyte with different concentration ranging from 0.01 to 0.5 M

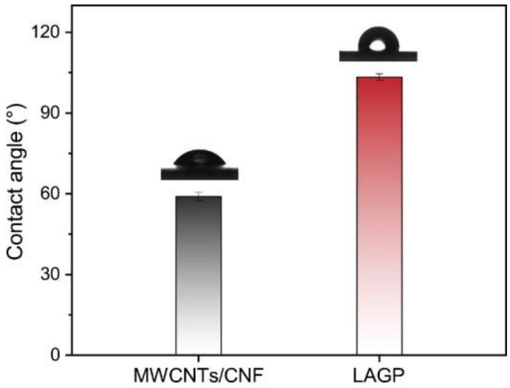

Fig. S3 Comparison of contact angle of MWCNTs/CNF (photothermal layer) and LAGP layer. Insert: corresponding water contact angles images. With the introduction of the photothermal layer, the contact angle is reduced from 103° to 59°. The increase in hydrophilicity facilitates ion transport of the proposed system.

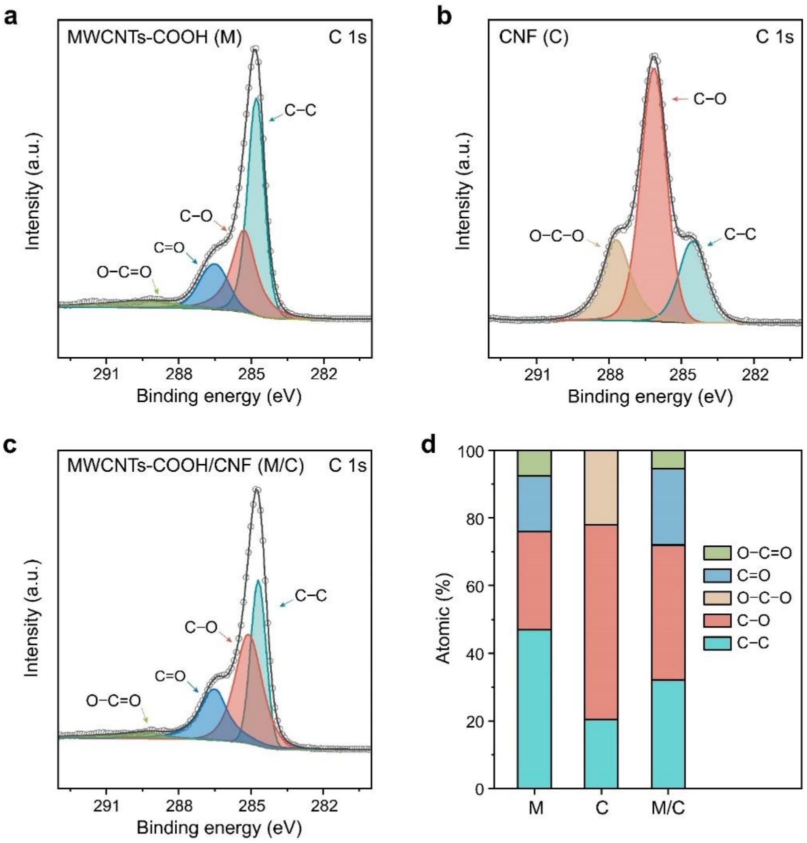

Fig. S4 C 1s XPS spectra of **a** MWCNTs-COOH, **b** CNF, **c** MWCNTs-COOH/CNF. **d** XPS peak positions data of C 1s. XPS spectra were deconvoluted into five peaks

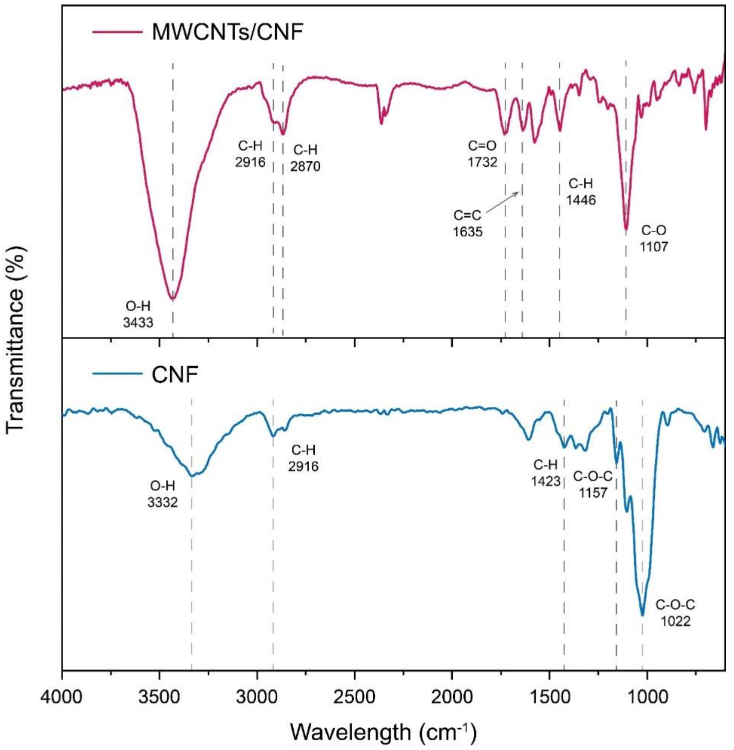

Fig. S5 Fourier transform infrared (FT-IR) spectrum of the MWCNTs/CNF and CNF indicating there are abundant carboxyl and hydroxyl groups within the photothermal layer

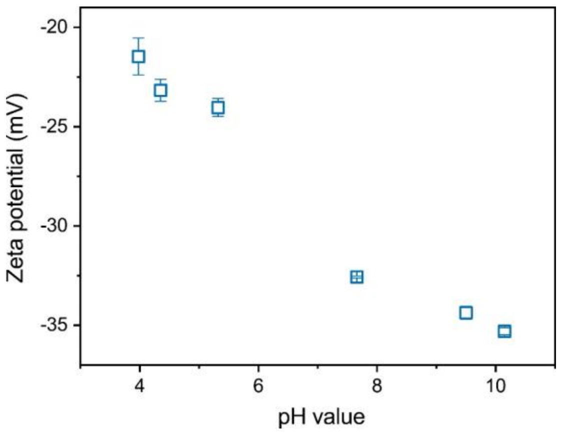

Fig. S6 Zeta potentials of the MWCNTs/CNF colloid (0.05 mg/mL) at various pH values, suggesting it was negatively charged in the whole pH range from 4 to 10

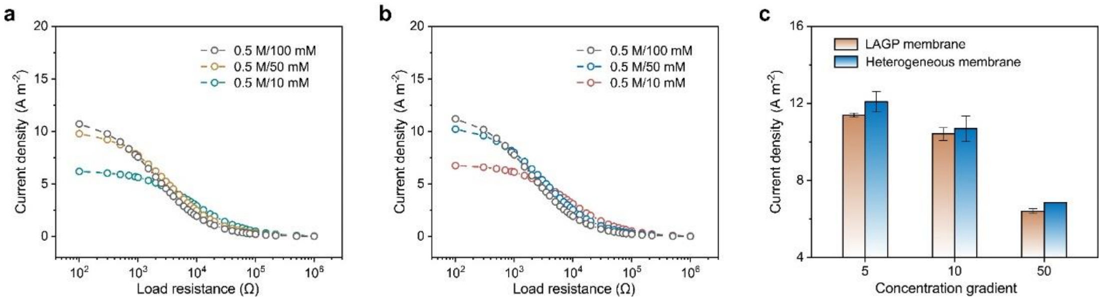

Fig. S7 Plot of current density as a function of load resistance in the transport system equipped with **a** LAGP membrane and **b** heterogeneous membrane. The harvested energy under a concentration gradient can be transferred to supply an external resistance. Under three concentration gradients, the measured current densities all gradually decrease with the increasing load resistance. **c** Comparison of current densities of LAGP and heterogeneous membranes at different concentrations. The current density of the heterogeneous membrane was greater than that of the LAGP membrane at 5-, 10- and 50-fold concentration gradients.

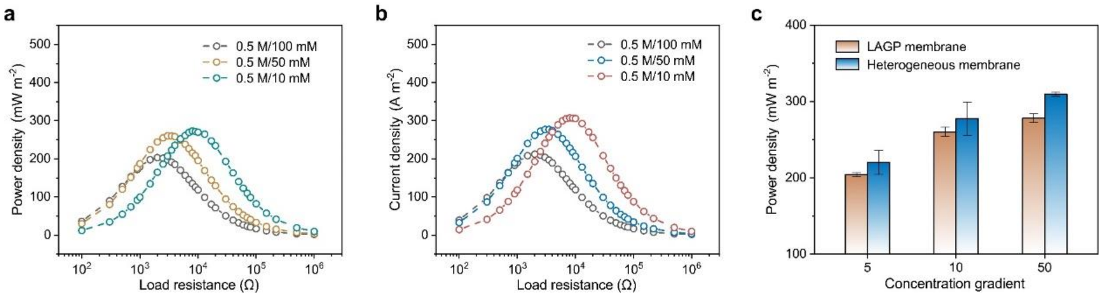

Fig. S8 Plot of power density as a function of load resistance in the transport system equipped with **a** LAGP membrane and **b** heterogeneous membrane. The harvested electric power of concentration gradient could be output to an external circuit for supplying an external load resistance (R_L). The output power density (P_R) consumed on R_L could be described as $P_R = I^2 R_L$, where I is the measured current at the corresponding R_L. As the external load resistance gradually increases, the current density decreases accordingly, but the output power density reaches a peak value at an intermediate external load resistance, respectively. **c** Comparison of power densities of LAGP and heterogeneous membranes at different concentrations. The power density of the heterogeneous membrane was greater than that of the LAGP membrane at 5-, 10- and 50-fold concentration gradients.

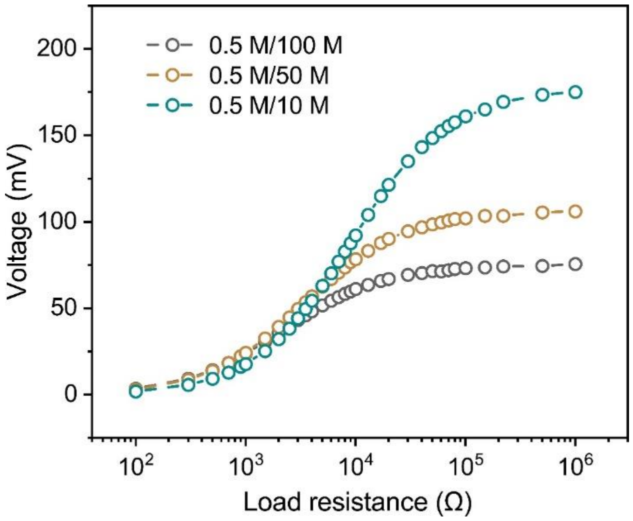

Fig. S9 Plot of voltage as a function of load resistance in the transport system equipped with LAGP membrane

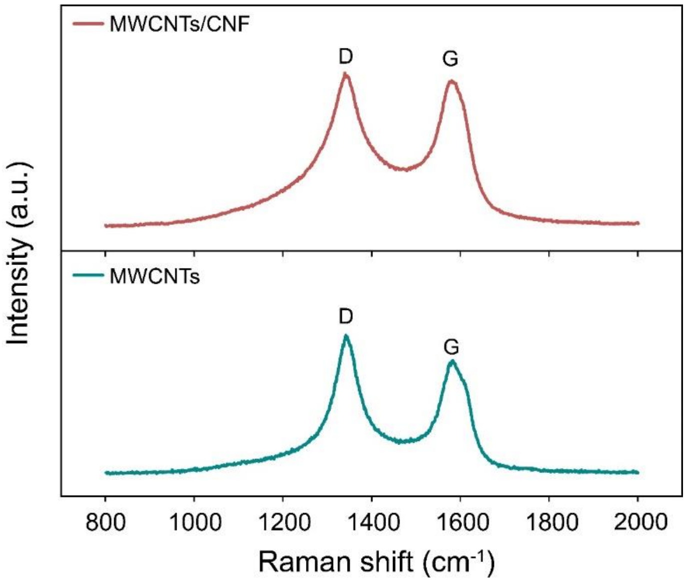

Fig. S10 Raman spectrum of MWCNTs/CNF and MWCNTs. The G band at approximately 1580 cm^{-1} originated from the in-plane vibration of sp^2 carbon atoms whereas the D bond at approximately 1340 cm^{-1} is attributed to the presence of disordered amorphous carbon (sp^3 carbon atoms), corresponding to double resonance effects in carbon.

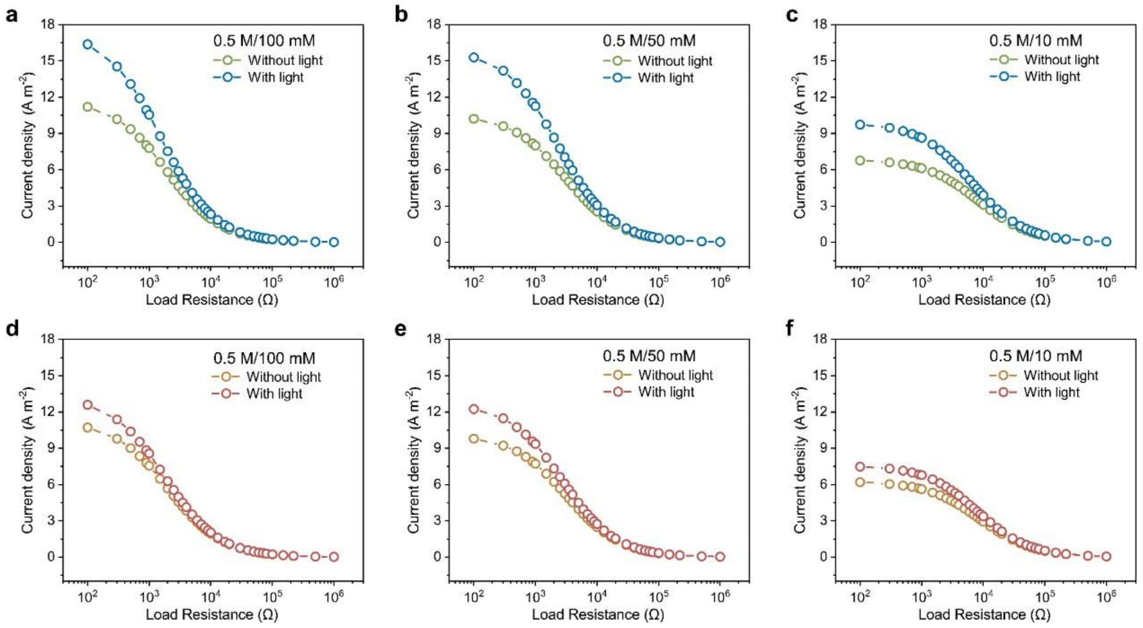

Fig. S11 Plot of current density of heterogeneous membrane as a function of load resistance with (blue line) and without (green line) light irradiation under **a** 5-fold concentration gradient, **b** 10-fold concentration gradient, and **c** 50-fold concentration gradient. Plot of current density of LAGP membrane as a function of load resistance with (red line) and without (orange line) light irradiation under **d** 5-fold concentration gradient, **e** 10-fold concentration gradient, and **f** 50-fold concentration gradient

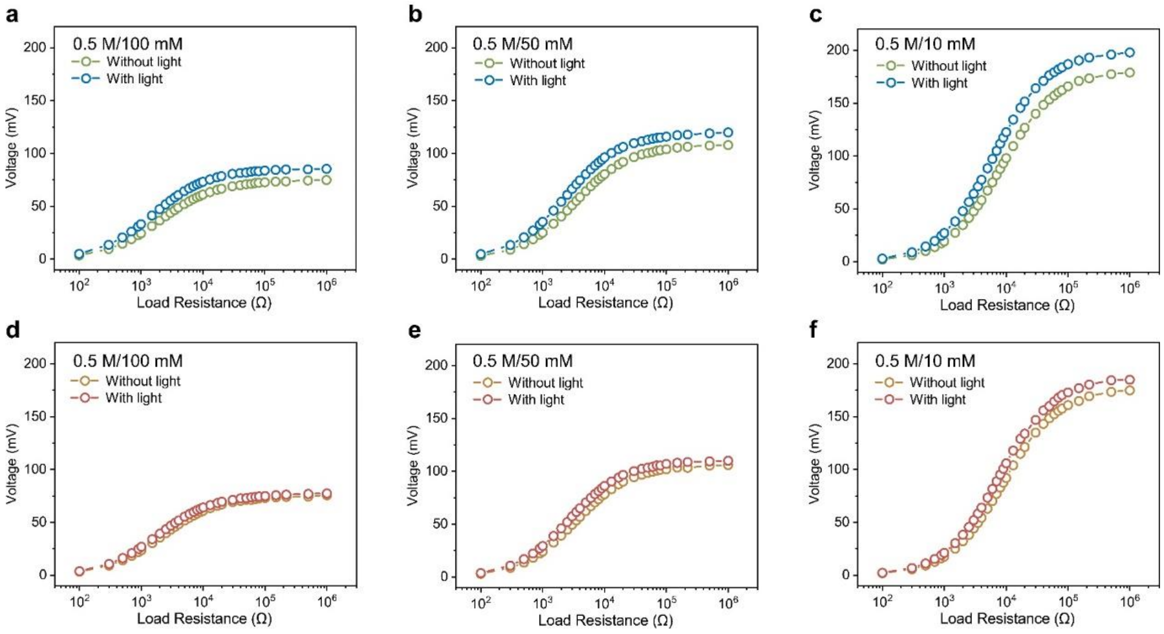

Fig. S12 Plot of voltage of heterogeneous membrane as a function of load resistance with (blue line) and without (green line) light irradiation under **a** 5-fold concentration gradient, **b** 10-fold concentration gradient, and **c** 50-fold concentration gradient. Plot of voltage of LAGP membrane as a function of load resistance with (red line) and without (orange line) light irradiation under **d** 5-fold concentration gradient, **e** 10-fold concentration gradient, and **f** 50-fold concentration gradient

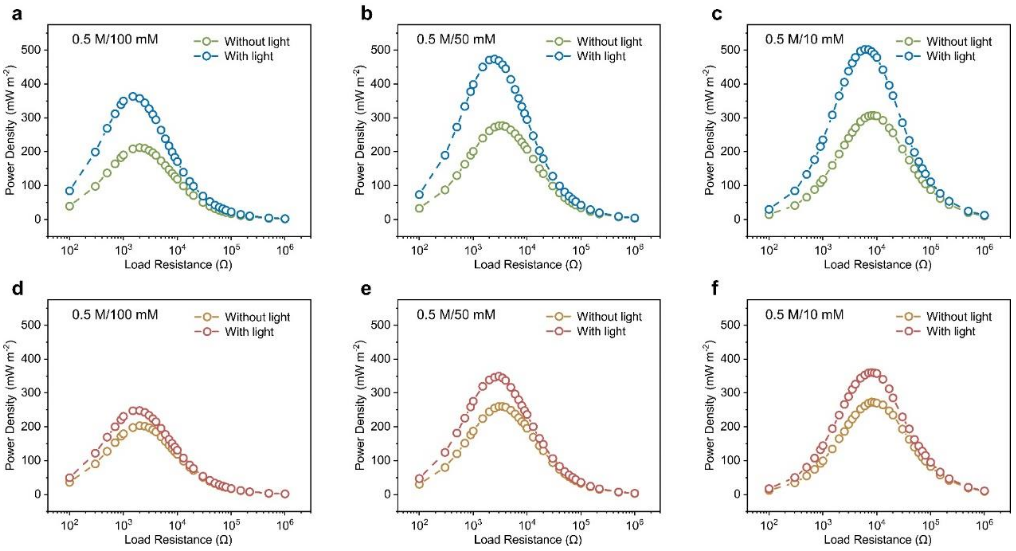

Fig. S13 Plot of power density of heterogeneous membrane as a function of load resistance with (blue line) and without (green line) light irradiation under **a** 5-fold concentration gradient, **b** 10-fold concentration gradient, and **c** 50-fold concentration gradient. Plot of power density of LAGP membrane as a function of load resistance with (red line) and without (orange line) light irradiation under **d** 5-fold concentration gradient, **e** 10-fold concentration gradient, and **f** 50-fold concentration gradient

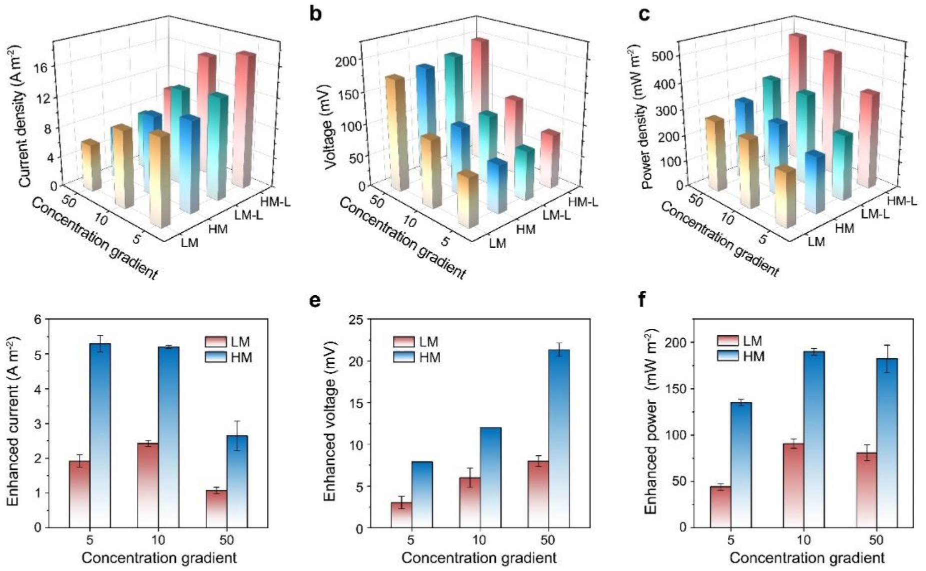

Fig. S14 a Current density comparison, **b** voltage comparison, and **c** power density comparison of LAGP membrane (LM), heterogeneous membrane (HM), LAGP membrane with light irradiation (LM-L), and heterogeneous membrane with light irradiation (HM-L) at concentration gradients of 5, 10 and 50, respectively. **d** Comparison of the enhanced current, **e** enhanced voltage, and **f** enhanced power of LM and HM induced by illumination. The current densities, voltages, and power densities of the HM in different concentration gradients were higher than those of bare LM in the absence of light conditions. When light is applied to the transport system, The current densities, voltages, and power densities of both HM and LM increase at different concentration gradients, while the enhancement of HM is significantly higher than that of LM due to the photothermal conversion effect of the photothermal layer in HM. It is indicated that the HM structure and the external field both have a prompting effect on ion transport.

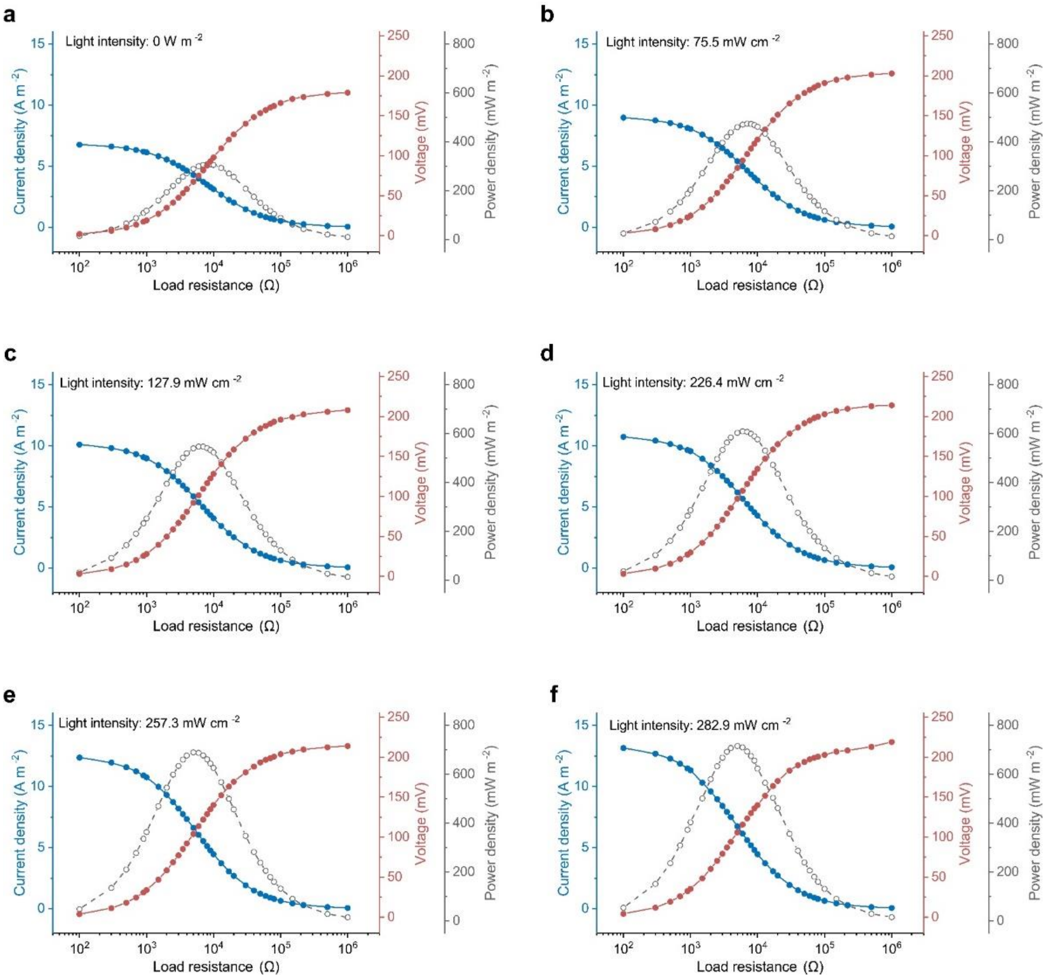

Fig. S15 Plot of current density (blue line), voltage (red line) and power density (gray line) of heterogeneous membrane as a function of load resistance under light irradiation with light intensity of **a** 0 mW cm^{-2}, **b** 75.5 mW cm^{-2}, **c** 127.9 mW cm^{-2}, **d** 226.4 mW cm^{-2}, **e** 257.3 mW cm^{-2}, **f** 282.9 mW cm^{-2}

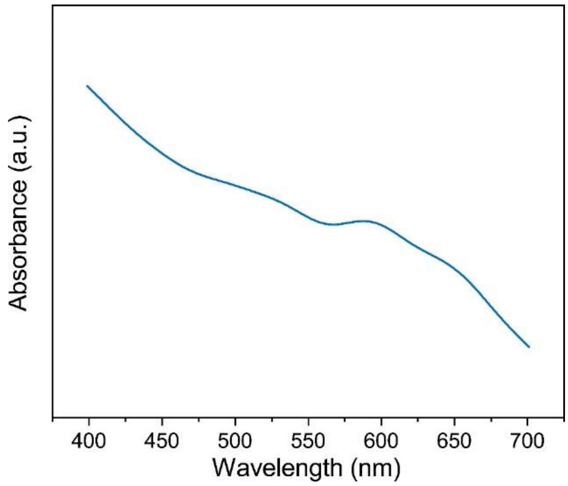

Fig. S16 UV-vis absorption spectra of MWCNTs/CNF

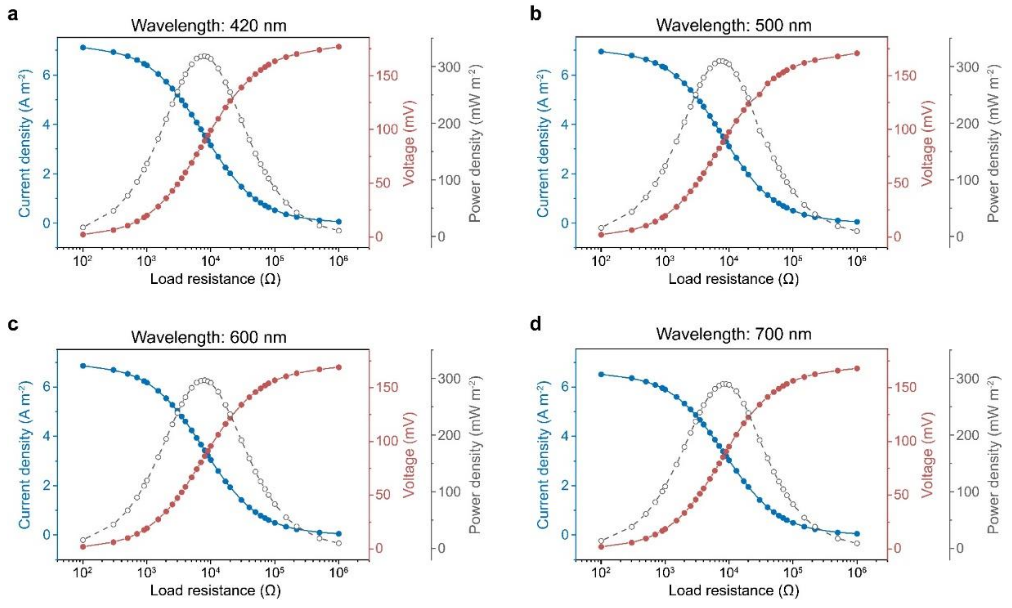

Fig. S17 Plot of current density (blue line), voltage (red line) and power density (gray line) of heterogeneous membrane as a function of load resistance under light irradiation with wavelength of **a** 420 nm, **b** 500 nm, **c** 600 nm, **d** 700 nm

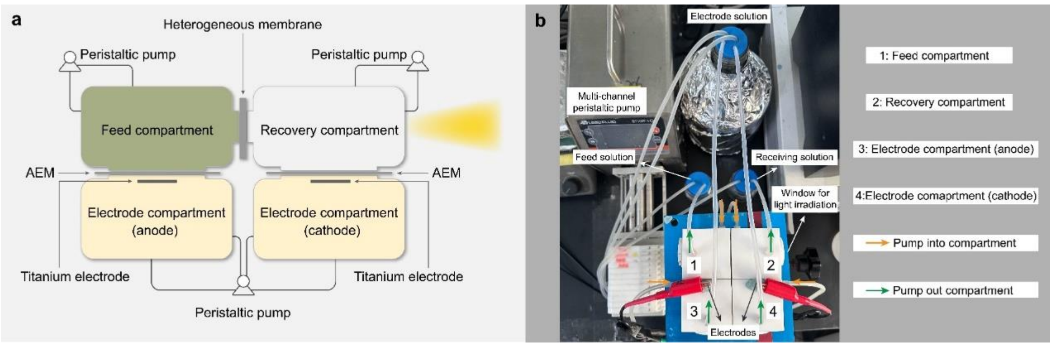

Fig. S18 a Schematic and **b** optical photo of the experimental setup used for Li-extraction from spent LIBs

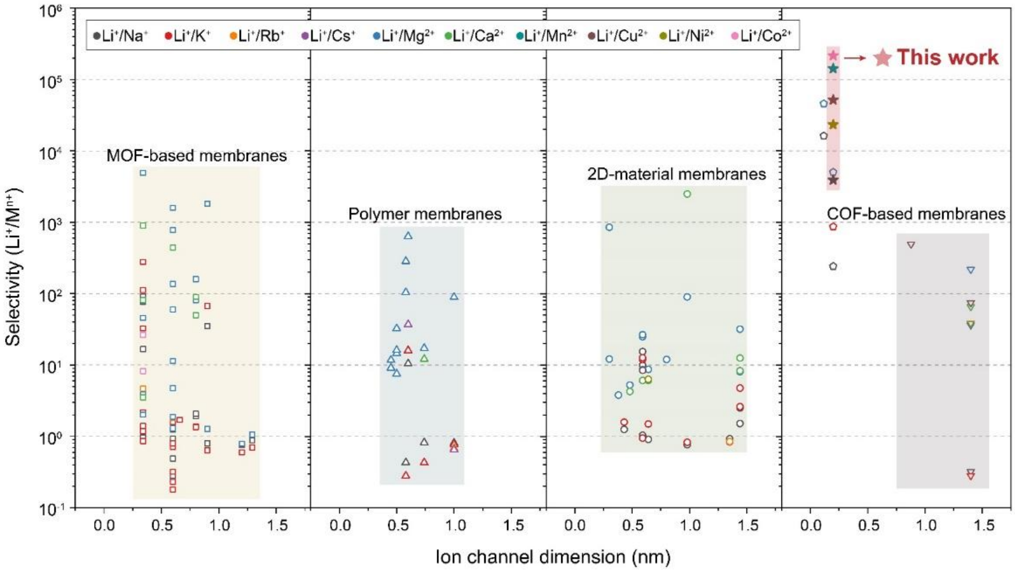

Fig. S19 Comparison of the lithium-ion selectivity with the reported porous membranes in the literature

S3 Supplementary Tables

Table S1 Chemical composition in the leaching solution of spent LIBs (feed solution)

Elements	Co	Cu	Li	Mn	Na	Ni
Concentration (g/L)	12.97	4.39	4.17	13.75	23.30	48.03

Table S2 Comparison of the Li^+/M^{x+} separation performance of this study with other membranes from literatures as shown in Fig. S19

**MOF-based membranes**	**Pore size (Å)**	**Ion Selectivity**	**Refs.**
MOP-18	6.6	Li^+/K^+=1.7	[S1]
Sulfonated HKUST-1	9.0	Li^+/Na^+=35; Li^+/K^+=67; Li^+/Mg^{2+}=1815	[S2]
HKUST-1@PVC	9.0	Li^+/Na^+=0.80; Li^+/K^+=0.64; Li^+/Mg^{2+}=1.27	[S3]
MOF-808@PVC	12.0	Li^+/Na^+=0.76; Li^+/K^+=0.6; Li^+/Mg^{2+}=0.79	[S3]
MOF-808-SO_3H@PVC	12.9	Li^+/Na^+=0.88; Li^+/K^+=0.70; Li^+/Rb^+=1.06	[S3]
ZIF-8	3.4	Li^+/Na^+=1.37; Li^+/K^+=2.18; Li^+/Mg^{2+}=4.67	[S4]
ZIF-8	3.4	Li^+/Na^+=0.97; Li^+/K^+=0.86; Li^+/Mg^{2+}=3.87; Li+/Ca^{2+}=3.52	[S5]
ZIF-8	3.4	Li^+/Na^+=1.3; Li^+/K^+=1.4; Li^+/Mg^{2+}=45.6	[S6]
SSP@ZIF-8	3.4	Li^+/Na^+=77; Li^+/K^+=112; Li^+/Mg^{2+}=4913	[S6]
ZIF-8@PVC	3.4	Li^+/Na^+=1.34; Li^+/K^+=1.18; Li^+/Mg^{2+}=2.02	[S3]
K-Carrageenan@ZIF-8	3.4	Li^+/Co^{2+}=8.23	[S7]
K-Carrageenan@ZIF-8	3.4	Li^+/Co^{2+}=26.39	[S7]
GZ-PIM3 (ZIF-8)	3.4	Li^+/Na^+=90.64; Li^+/K^+=278.39; Li^+/Mg^{2+}=893.75; Li+/Ca^{2+}=898.34	[S8]
CA-PIM (ZIF-8)	3.4	Li^+/Na^+=16.7; Li^+/K^+=32.6; Li^+/Mg^{2+}=78.67; Li+/Ca^{2+}=81.2	[S8]
UiO-67	8.0	Li^+/Na^+=1.92; Li^+/K^+=1.37; Li^+/Mg^{2+}=87; Li+/Ca^{2+}=50	[S9]
UiO-67	8.0	Li^+/Na^+=2.05; Li^+/K^+=1.35; Li^+/Mg^{2+}=159.4; Li+/Ca^{2+}=90	[S9]
UiO-66	6.0	Li^+/Na^+=1.24; Li^+/K^+=1.58; Li^+/Rb^+=1.82	[S4]
UiO-66@PVC	6.0	Li^+/Na^+=0.84; Li^+/K^+=0.71; Li^+/Mg^{2+}=1.3	[S3]
UiO-66-HSO_3@PVC	6.0	Li^+/Na^+=0.93; Li^+/K^+=0.8; Li^+/Mg^{2+}=4.79	[S3]
UiO-66-HSO_3@PVC	6.0	Li^+/Mg^{2+}=4.73	[S3]

UiO-66-NH_2	6.0	Li^+/Mg^{2+}=60	[S10]
UiO-66-SO_3H	6.0	Li^+/Mg^{2+}=1.88	[S11]
UiO-66-SO_3H	6.0	Li^+/Na^+=0.32Li^+/K^+=0.18 Li^+/Mg^{2+}=776	[S11]
UiO-66(Zr/Ti)-NH_2@Polyamide	6.0	Li^+/Mg^{2+}=11.38	[S12]
UiO-66-$(COOH)_2$	6.0	Li^+/Na^+=0.49; Li^+/K^+=0.32; Li^+/Mg^{2+}=1590.1; Li^+/Ca^{2+}=441.7	[S13]
UiO-66-COOH	6.0	Li^+/Na^+=0.24; Li^+/K^+=0.18; Li^+/Mg^{2+}=136.5	[S14]
UiO-66-NH_2	6.0	Li^+/Na^+=0.28; Li^+/K^+=0.23; Li^+/Mg^{2+}=1.3	[S14]
**Polymer membranes**	**Pore size (Å)**	**Ion Selectivity**	**Refs.**
PET	6	Li^+/Na^+=10.46; Li^+/K^+=16.02; Li^+/Cs^+=37.09;Li^+/Mg^{2+}=633.98	[S15]
PET	10	Li^+/Na^+=0.81; Li^+/K^+=0.77; Li^+/Rb^+=0.68	[S15]
Porous organic cage membrane (CC3)	5.8	Li^+/Na^+=0.43; Li^+/K^+=0.28; Li^+/Mg^{2+}=284	[S16]
Porous organic cage membrane (CC3)	5.8	Li^+/Mg^{2+}=104	[S16]
Conjugated microporous polymer membrane (CMP)	7.4	Li^+/Na^+=0.82; Li^+/K^+=0.43; Li^+/Mg^{2+}=17.2; Li^+/Ca^{2+}=12.1	[S17]
HMO@SPES	4.5	Li^+/Mg^{2+}=9.1	[S18]
HMO@SPES	4.5	Li^+/Mg^{2+}=11.75	[S18]
PIM-EA-TB	~5.0	Li^+/Mg^{2+}=7.53	[S19]
PIM-BzMA-TB	~5.0	Li^+/Mg^{2+}=32.6	[S19]
DMBP-TB	~5.0	Li^+/Mg^{2+}=14.6	[S19]
AO-PIM-1	~5.0	Li^+/Mg^{2+}=16.1	[S19]
**2D-material membranes**	**Pore size (Å)**	**Ion Selectivity**	**Refs.**
GO	13.5	Li^+/Na^+=0.93; Li^+/K^+=0.84; Li^+/Rb^+=0.85	[S4]
GO	9.8	Li^+/Na^+=0.77; Li^+/K^+=0.93; Li^+/Mg^{2+}=~90; Li^+/Ca^{2+}=~2500	[S20]
Sulfonated-rGO	4.8	Li^+/Mg^{2+}=5.27; Li^+/Ca^{2+}=4.27	[S21]

Vermiculite	4.3	Li^+/Na^+=1.26; Li^+/K^+=1.59	[S22]
MXene	6.4	Li^+/Na^+=0.97; Li^+/K^+=1.5; Li^+/Mg^{2+}=8.75; Li^+/Ca^{2+}=6.09; Li^+/Ni^{2+}=6.36	[S23]
MXene@PSS	5.9	Li^+/Na^+=10; Li^+/K^+=12; Li^+/Mg^{2+}=25	[S24]
MXene@PSS	5.9	Li^+/Na^+=15.5; Li^+/K^+=12.7; Li^+/Mg^{2+}=26.7	[S24]
VMT	~3.0	Li^+/Mg^{2+}=12.2	[S25]
VMT	~3.0	Li^+/Mg^{2+}=856	[S25]
i-GO	~5.9	Li^+/Na^+=1.04; Li^+/K^+=0.95; Li^+/Mg^{2+}=8.64; Li^+/Ca^{2+}=6.11; Li^+/Cu^{2+}=8.47	[S26]
GO/MXene	14.4	Li^+/Na^+=2.52; Li^+/K^+=4.78; Li^+/Mg^{2+}=8.07; Li^+/Ca^{2+}=8.36	[S27]
GO/MXene	14.4	Li^+/Na^+=1.52; Li^+/K^+=2.62; Li^+/Mg^{2+}=31.9; Li^+/Ca^{2+}=12.59	[S27]
rGO@SAPS	3.8	Li^+/Mg^{2+}=3.8	[S28]
FRGO	8.0	Li^+/Mg^{2+}=~12	[S29]
**COF-based membranes**	**Pore size (Å)**	**Ion Selectivity**	**Refs.**
TpBDMe2 COF	14	Li^+/Mg^{2+}=36	[S30]
TpBDMe2 COF	14	Li^+/Na^+=0.32; Li^+/K^+=0.28; Li^+/Mg^{2+}=217; Li^+/Ca^{2+}=65.4; Li^+/Cu^{2+}=74.1; Li^+/Ni^{2+}=38.4	[S30]
3D-OH-COF	8.8	Li^+/Cu^{2+}=490	[S31]
COF-EB_1BD_1	2	Li^+/Mg^{2+}=443	[S32]
**Other membranes**	**Pore size (Å)**	**Ion Selectivity**	**Refs.**
LAGP/(MWCNTs-NH_2/PEDOT:PSS)	0.2	Li^+/Na^+=242; Li^+/K^+=870; Li^+/Mg^{2+}=5032	[S33]
LLTO	0.118	Li^+/Na^+=16227; Li^+/Mg^{2+}=45916	[S34]
LAGP/MWCNTs	0.2	Li^+/Na^+=3907; Li^+/Mn^{2+}=142043; Li^+/Co^{2+}=216412; Li^+/Ni^{2+}=23276; Li^+/Cu^{2+}=51843	This work

Supplementary References

[S1] M. Jung, H. Kim, K. Baek, K. Kim, Synthetic ion channel based on metal-organic polyhedra. Angew. Chem. Int. Ed. **47**(31), 5755-5757 (2008). https://doi.org/10.1002/anie.200802240

[S2] Y. Guo, Y. Ying, Y. Mao, X. Peng, B. Chen, Polystyrene sulfonate threaded through a metal-organic framework membrane for fast and selective lithium-ion separation. Angew. Chem. Int. Ed. **55**(48), 15120-15124 (2016). https://doi.org/10.1002/anie.201607329

[S3] C. Zhang, Y. Mu, W. Zhang, S. Zhao, Y. Wang, PVC-based hybrid membranes containing metal-organic frameworks for Li^+/Mg^{2+} separation. J. Membr. Sci. **596**, 117724 (2020). https://doi.org/10.1016/j.memsci.2019.117724

[S4] H. Zhang, J. Hou, Y. Hu, P. Wang, R. Ou et al., Ultrafast selective transport of alkali metal ions in metal organic frameworks with subnanometer pores. Sci. Adv. **4**(2), eaaq0066 (2018). https://doi.org/10.1126/sciadv.aaq0066

[S5] M. Mohammad, M. Lisiecki, K. Liang, A. Razmjou, V. Chen, Metal-phenolic network and metal-organic framework composite membrane for lithium ion extraction. Appl. Mater. Today. **21**, 100884 (2020). https://doi.org/10.1016/j.apmt.2020.100884

[S6] H.-Q. Liang, Y. Guo, X. Peng, B. Chen, Light-gated cation-selective transport in metal-organic framework membranes. J. Mater. Chem. A **8**(22), 11399-11405 (2020). https://doi.org/10.1039/D0TA02895A

[S7] Z. Li, Y. Guo, X. Wang, P. Li, W. Ying et al., Simultaneous recovery of metal ions and electricity harvesting via k-carrageenan@ZiF-8 membrane. ACS Appl. Mater. Interfaces **11**(37), 34039-34045 (2019). https://doi.org/10.1021/acsami.9b12501

[S8] H. Kazemzadeh, J. Karimi-Sabet, J. Towfighi Darian, A. Adhami, Evaluation of polymer inclusion membrane efficiency in selective separation of lithium ion from aqueous solution. Sep. Purif. Technol. **251**(117298 (2020). https://doi.org/10.1016/j.seppur.2020.117298

[S9] R. Xu, Y. Kang, W. Zhang, X. Zhang, B. Pan, Oriented UiO-67 metal-organic framework membrane with fast and selective lithium-ion transport. Angew. Chem. Int. Ed. **61**(3), e202115443 (2022). https://doi.org/https://doi.org/10.1002/anie.202115443

[S10] T. Xu, M. A. Shehzad, D. Yu, Q. Li, B. Wu et al., Highly cation permselective metal-organic framework membranes with leaf-like morphology. ChemSusChem. **12**(12), 2593-2597 (2019). https://doi.org/10.1002/cssc.201900706

[S11] T. Xu, M. A. Shehzad, X. Wang, B. Wu, L. Ge et al., Engineering leaf-like UiO-66-SO_3H membranes for selective transport of cations. Nano-Micro Lett. **12**(1), 51 (2020). https://doi.org/10.1007/s40820-020-0386-6

[S12] T. Xu, F. Sheng, B. Wu, M. A. Shehzad, A. Yasmin et al., Ti-exchanged UiO-66-NH_2-containing polyamide membranes with remarkable cation permselectivity. J. Membr. Sci. **615**, 118608 (2020).

https://doi.org/10.1016/j.memsci.2020.118608

[S13] J. Lu, H. Zhang, J. Hou, X. Li, X. Hu et al., Efficient metal ion sieving in rectifying subnanochannels enabled by metal-organic frameworks. Nat. Mater. **19**(7), 767-774 (2020). https://doi.org/10.1038/s41563-020-0634-7

[S14] J. Lu, H. Zhang, X. Hu, B. Qian, J. Hou et al., Ultraselective monovalent metal ion conduction in a three-dimensional sub-1 nm nanofluidic device constructed by metal-organic frameworks. ACS Nano. **15**(1), 1240-1249 (2021). https://doi.org/10.1021/acsnano.0c08328

[S15] Q. Wen, D. Yan, F. Liu, M. Wang, Y. Ling et al., Highly selective ionic transport through subnanometer pores in polymer films. Adv. Funct. Mater. **26**(32), 5796-5803 (2016). https://doi.org/10.1002/adfm.201601689

[S16] T. Xu, B. Wu, L. Hou, Y. Zhu, F. Sheng et al., Highly ion-permselective porous organic cage membranes with hierarchical channels. J. Am. Chem. Soc. **144**(23), 10220-10229 (2022). https://doi.org/10.1021/jacs.2c00318

[S17] Z. Zhou, D. B. Shinde, D. Guo, L. Cao, R. A. Nuaimi et al., Flexible ionic conjugated microporous polymer membranes for fast and selective ion transport. Adv. Funct. Mater. **32**(6), 2108672 (2022). https://doi.org/10.1002/adfm.202108672

[S18] H. M. Saif, R. M. Huertas, S. Pawlowski, J. G. Crespo, S. Velizarov, Development of highly selective composite polymeric membranes for Li^+/Mg^{2+} separation. J. Membr. Sci. **620**, 118891 (2021). https://doi.org/10.1016/j.memsci.2020.118891

[S19] R. Tan, A. Wang, R. Malpass-Evans, R. Williams, E. W. Zhao et al., Hydrophilic microporous membranes for selective ion separation and flow-battery energy storage. Nat. Mater. **19**(2), 195-202 (2020). https://doi.org/10.1038/s41563-019-0536-8

[S20] J. Abraham, K. S. Vasu, C. D. Williams, K. Gopinadhan, Y. Su et al., Tunable sieving of ions using graphene oxide membranes. Nat. Nanotechnol. **12**(6), 546-550 (2017). https://doi.org/10.1038/nnano.2017.21

[S21] Y. Zhao, W. Shi, B. Van der Bruggen, C. Gao, J. Shen, Tunable nanoscale interlayer of graphene with symmetrical polyelectrolyte multilayer architecture for lithium extraction. Adv. Mater. Interfaces **5**(6), 1701449 (2018). https://doi.org/10.1002/admi.201701449

[S22] A. Razmjou, G. Eshaghi, Y. Orooji, E. Hosseini, A. H. Korayem et al., Lithium ion-selective membrane with 2d subnanometer channels. Water Res. **159**, 313-323 (2019). https://doi.org/10.1016/j.watres.2019.05.018

[S23] C. E. Ren, K. B. Hatzell, M. Alhabeb, Z. Ling, K. A. Mahmoud et al., Charge- and size-selective ion sieving through $ti_3c_2t_x$ mxene membranes. J. Phys. Chem. Lett. **6**(20), 4026-4031 (2015). https://doi.org/10.1021/acs.jpclett.5b01895

[S24] Z. Lu, Y. Wu, L. Ding, Y. Wei, H. Wang, A lamellar MXene ($ti_3c_2t_x$)/pss composite membrane for fast and selective lithium-ion separation. Angew. Chem. Int. Ed. **60**(41), 22265-22269 (2021). https://doi.org/10.1002/anie.202108801

[S25] T. Zhang, H. Bai, Y. Zhao, B. Ren, T. Wen et al., Precise cation recognition in two-dimensional nanofluidic channels of clay membranes imparted from intrinsic selectivity of clays. ACS Nano **16**(3), 4930-4939 (2022). https://doi.org/10.1021/acsnano.2c00866

[S26] M. Zhang, P. Zhao, P. Li, Y. Ji, G. Liu et al., Designing biomimic two-dimensional ionic transport channels for efficient ion sieving. ACS Nano **15**(3), 5209-5220 (2021). https://doi.org/10.1021/acsnano.0c10451

[S27] W. Xin, C. Lin, L. Fu, X.-Y. Kong, L. Yang et al., Nacre-like mechanically robust heterojunction for lithium-ion extraction. Matter **4**(2), 737-754 (2021). https://doi.org/10.1016/j.matt.2020.12.003

[S28] Y. Zhao, C. Zhou, J. Wang, H. Liu, Y. Xu et al., Formation of morphologically confined nanospaces via self-assembly of graphene and nanospheres for selective separation of lithium. J. Mater. Chem. A **6**(39), 18859-18864 (2018). https://doi.org/10.1039/C8TA06945J

[S29] Y.-H. Xi, Z. Liu, J. Ji, Y. Wang, Y. Faraj et al., Graphene-based membranes with uniform 2D nanochannels for precise sieving of mono-/multi-valent metal ions. J. Membr. Sci. **550**, 208-218 (2018). https://doi.org/10.1016/j.memsci.2017.12.057

[S30] F. Sheng, B. Wu, X. Li, T. Xu, M. A. Shehzad et al., Efficient ion sieving in covalent organic framework membranes with sub-2-nanometer channels. Adv. Mater. **33**(44), 2104404 (2021). https://doi.org/10.1002/adma.202104404

[S31] X. Shi, Z. Zhang, S. Fang, J. Wang, Y. Zhang et al., Flexible and robust three-dimensional covalent organic framework membranes for precise separations under extreme conditions. Nano Lett. **21**(19), 8355-8362 (2021). https://doi.org/10.1021/acs.nanolett.1c02919

[S32] L. Hou, W. Xian, S. Bing, Y. Song, Q. Sun et al., Understanding the ion transport behavior across nanofluidic membranes in response to the charge variations. Adv. Funct. Mater. **31**(16), 2009970 (2021). https://doi.org/10.1002/adfm.202009970

[S33] L. Fu, Y. Teng, P. Liu, W. Xin, Y. Qian et al., Electrochemical ion-pumping-assisted transfer system featuring a heterogeneous membrane for lithium recovery. Chem. Eng. J. **435**, 134955 (2022). https://doi.org/10.1016/j.cej.2022.134955

[S34] Z. Li, C. Li, X. Liu, L. Cao, P. Li et al., Continuous electrical pumping membrane process for seawater lithium mining. Energy Environ. Sci. **14**(5), 3152-3159 (2021). https://doi.org/10.1039/D1EE00354B
